# Nephroprotective effects of substances of medicine food homology and traditional Chinese medicine phytochemicals against acute kidney injury

**DOI:** 10.3389/fphar.2025.1539886

**Published:** 2025-02-19

**Authors:** Ling Chen, Yanyan Deng, Jing Hu, Xuezhong Gong

**Affiliations:** ^1^ Department of Nephrology, Seventh People’s Hospital of Shanghai University of Traditional Chinese Medicine, Shanghai, China; ^2^ Shanghai Frontiers Science Center of TCM Chemical Biology, Institute of Interdisciplinary Integrative Medicine Research, Shanghai University of Traditional Chinese Medicine, Shanghai, China; ^3^ Department of Nephrology, Shanghai Municipal Hospital of Traditional Chinese Medicine, Shanghai University of Traditional Chinese Medicine, Shanghai, China

**Keywords:** nephroprotective effects, substances of medicine food homology, traditional Chinese medicine, phytochemicals, acute kidney injury

## Abstract

Acute kidney injury (AKI) represents significant medical challenges due to its elevated rates of morbidity and mortality, with limited therapeutic options currently available. Hence, the exploration of novel medicinal treatments for AKI management remains vital. Substances of medicine food homology (SMFH), referring to substances having characteristics of both food and medicine, have been applied in China for thousands years.They could be used for daily diets and body conditioning. Traditional Chinese medicine (TCM), with its naturally derived components and demonstrated effectiveness, presents distinctive benefits in AKI treatment. Numerous studies have shown that SMFH and TCM phytochemicals could function satisfactorily with nephroprotective effects and have a significant effect on alleviating AKI as well as its complications. In this review, the pathogenesis of AKI was illustrated. We concentrated on SMFH and TCM phytochemicals against AKI and tried to summarize the underlying mechanisms in various kinds of AKI, highlighting the crucial phytochemical components in AKI prevention and therapy. Besides, strategies for SMFH and TCM phytochemicals globalization are analysed. This review comprehensively reveals that SMFH and TCM phytochemicals exhibit promising potential for AKI intervention by targeting various signal pathways and targets, which would contribute to AKI’s cognition, preventive treatments, as well as global promotion.

## 1 Introduction

Acute kidney injury (AKI) represents a critical medical condition linked to rising morbidity and mortality rates, affecting 10%–15% of hospitalized individuals, with up to 50% of those requiring admission to intensive care units (Ronco et al., 2019; Neyra et al., 2023; Xu et al., 2024). This condition ranks among the most prevalent critical illnesses, characterized by a reduced glomerular filtration rate, accumulation of nitrogenous waste products, disturbances in water and electrolyte balance, and acid-base imbalance as its primary clinical features. Currently, no effective treatment exists for AKI; however, the primary approach involves addressing the underlying diseases, eliminating risk elements, sustaining acid-base homeostasis and water-electrolyte equilibrium, along with renal replacement therapy. Despite these interventions, the mortality rate remains considerably high (Allinson et al., 2023; Du et al., 2023). Survivors of AKI are notably predisposed to chronic kidney disease (CKD) and end-stage renal disease (ESRD) (Tao et al., 2024). Clinically, AKI may arise from a variety of causes, encompassing sepsis, ischemia/reperfusion (I/R) injury, and exposure to different nephrotoxin, among others (Chen and Gong, 2022; Chen et al., 2024a). The treatment of AKI primarily emphasizes supportive interventions, encompassing optimizing hemodynamic volume status and minimizing nephrotoxic exposure. Apart from conservative management, there are presently no medical or surgical alternatives available for the prevention or treatment of AKI other than renal replacement therapy (Vijayan, 2021; Shi et al., 2023). AKI is recognized as a worldwide healthcare concern, thus highlighting the urgent need to identify novel therapeutic approaches and agents for managing this condition (Song and Gong, 2023).

Substances of medicine food homology (SMFH), referring to substances having characteristics of both food and medicine, have been applied in China for thousands years (Chen, 2023). As an integral and important part of traditional Chinese medicine (TCM), they could be used for daily diets and body conditioning (Qu et al., 2023; Liu H. et al., 2024). Over the past few years, TCM has experienced growing acceptance worldwide for treating AKI, attributed to its comprehensive dialectical system and remarkable clinical efficacy (Chen et al., 2022; Song et al., 2023; Chen et al., 2024b). Investigations into the use of TCM and its compounds for treating AKI have gradually intensified. These treatments are characterized by their numerous ingredients, diverse targets, and distinct mechanisms, possibly providing special clinical benefits in AKI prevention and control (Zhong et al., 2023). Phytochemicals, the bioactive compounds within TCM, exhibit potential in preventing and managing AKI via multiple pathways, encompassing reducing oxidative stress (OS), modulating autophagy processes, suppressing inflammation, and alleviating damage to mitochondria (Shi et al., 2023). However, a systematic review of SMFH and TCM phytochemicals addressing various types of AKI remains lacking. This review summarizes the existing data supporting the capacity of SMFH and TCM phytochemicals to enhance AKI outcomes and provides both clinical and experimental evidence relevant to AKI management. Additionally, strategies for SMFH and TCM phytochemicals globalization are analysed for their global promotion.

## 2 Literature search strategies

The PubMed database was chosen due to its exceptional precision in document classification, rendering it the most suitable platform for bibliometric evaluation. A literature exploration was conducted on 12 May 2024, through PubMed to identify publications addressing the application of TCM botanical compounds in relation to AKI spanning from 12 May 2014, to 12 May 2024. The database investigation employed the following criteria (TS = (Acute Kidney Injury) OR TS = (Acute Renal Injury) OR TS = (Acute Renal Insufficiency) OR TS = (Acute Kidney Insufficiency)) AND (TS = (traditional Chinese medicine) OR TS = (Herbal Medicine) OR TS = (Chinese medicine) OR TS = (Chinese herbs) OR TS = (Substances of medicine food homology) OR TS = (Phytochemicals)). Contents include reviews, clinical trials, *in vivo or in vitro* experiments, *etc.*, 140 articles were selected for discussion after filtering the search results and excluding non-relevant literature. All findings are depicted in the form of narrative reviews within this document.

## 3 The pathogenesis of AKI

**FIGURE 1:**
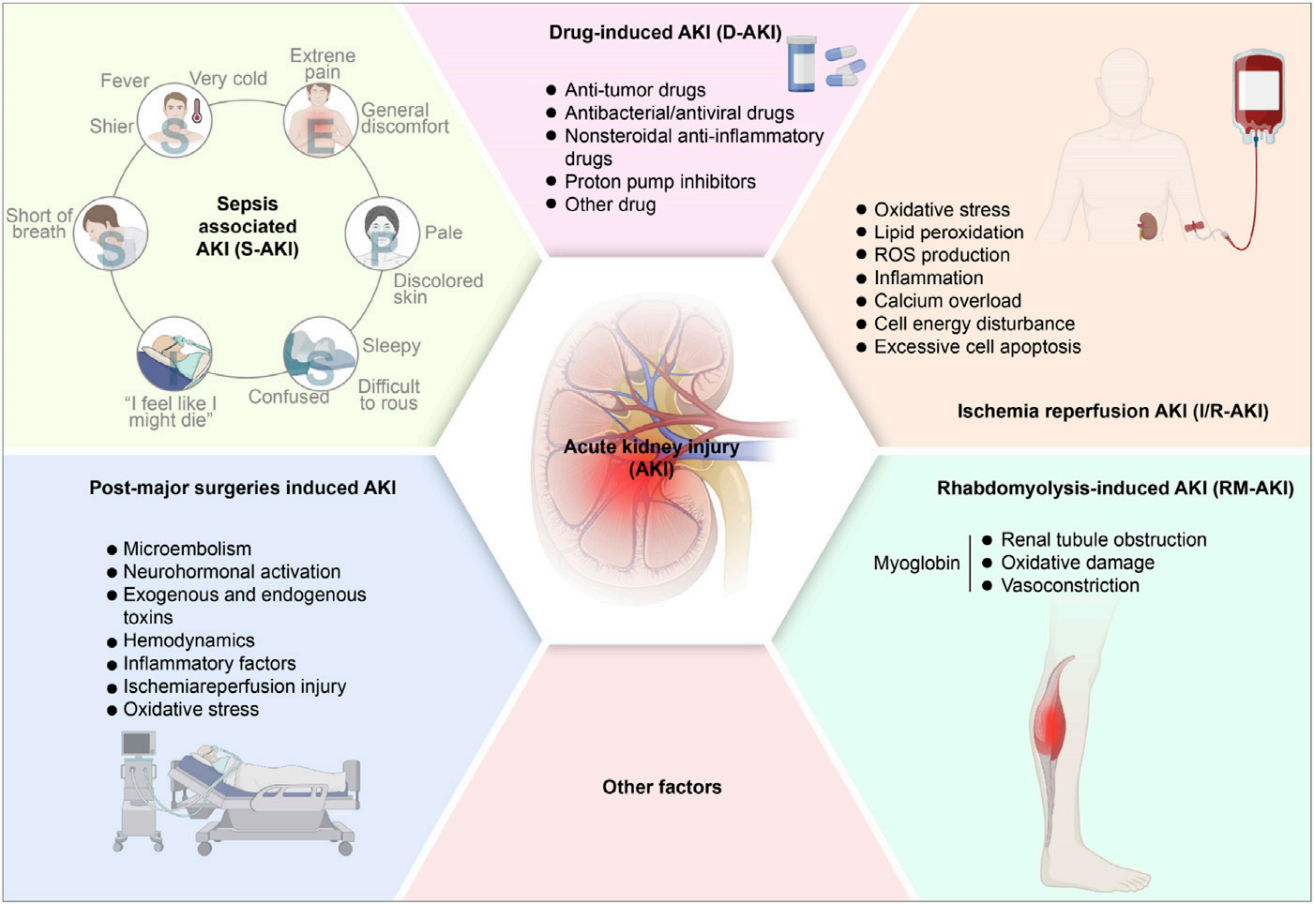
The pathogenesis of acute kidney injury. AKI, acute kidney injury; S-AKI, Sepsis associated acute kidney injury; D-AKI, drug-induced acute kidney injury; I/R-AKI, Ischemia-reperfusion acute kidney injury; RM-AKI, Rhabdomyolysis-induced acute kidney injury.

The primary factors leading to AKI encompass sepsis, nephrotoxic agents, renal I/R injury, rhabdomyolysis (RM), post-major surgeries, etc (Bellomo et al., 2017; Tang C. et al., 2023; Yang W. et al., 2023). The pathogenesis of different types of AKI varies according to the different causes. AKI could be divided into the following categories (Figure 1).

### 3.1 Sepsis-associated AKI (S-AKI)

Sepsis, characterized as a systemic inflammatory response syndrome associated with infection, frequently leads to multiple organ dysfunction, particularly impacting the kidneys and resulting in sepsis-associated AKI (S-AKI). S-AKI is regarded as the most common complication of sepsis (Peerapornratana et al., 2019). Generally, sepsis is responsible for 45%–70% of AKI occurrences in critically ill individuals (Zarbock et al., 2023), correlating with extended stays in hospitals, elevated mortality rates, an increased incidence of long-term disabilities, and a reduced quality of life (Poston and Koyner, 2019; White et al., 2023). Importantly, the pathophysiology of sepsis is intricate and distinct, rendering S-AKI unique among other AKI phenotypes (Poston and Koyner, 2019). Lipopolysaccharides (LPS) are pivotal in the pathogenesis of sepsis. The underlying mechanisms contributing to S-AKI are multifaceted, encompassing alterations in microcirculation, complex inflammatory pathways, and cellular demise (Chang et al., 2022). To date, various patterns of cell death, encompassing apoptosis, necrosis, necroptosis, and autophagy, have been identified as significant in S-AKI (Wu Z. et al., 2022). The mechanisms underlying LPS-induced AKI may involve inflammation, renal I/R injury, OS, and systemic hypotension. The inflammatory and oxidative pathways present possible therapeutic interventions for addressing AKI during septic conditions (Cao et al., 2024). Consequently, addressing S-AKI through preventive measures and treatment protocols, as well as the reduction of morbidity and mortality among septic patients, represent critical public health challenges.

### 3.2 Drug-induced AKI (D-AKI)

Drug-induced AKI (D-AKI) represents a serious adverse event, constituting roughly 20% of AKI incidents (Hosohata, 2016; Perazella and Rosner, 2022). Frequently encountered nephrotoxic substances include anticancer medications (notably cisplatin and doxorubicin), antimicrobial and antiviral compounds (like gentamicin), Non-Steroidal Antiinflammatory Drugs (NSAIDs), contrast media, and proton pump blockers. While cisplatin stands as a potent chemotherapeutic compound, its therapeutic application faces constraints owing to kidney toxicity. Cisplatin-induced AKI (Cis-induced AKI) exhibits strong connections with DNA harm, OS, and inflammation (Guo et al., 2022; Xu et al., 2023). The involvement of various regulated cell death mechanisms, encompassing ferroptosis, in Cis-induced AKI, has only recently gained recognition (Kim et al., 2022). In recent years, relevant studies (Gong et al., 2015; Gong et al., 2019; Li and Gong, 2022) have shown that contrast agents-induced AKI also account for a considerable proportion. Its mechanism of action is mainly due to the nephrotoxic effect of contrast agents on renal tubules and vascular endothelial cells, which leads to hemodynamic changes, OS, apoptosis, and inflammatory reactions in the kidney.

### 3.3 I/R-induced AKI (I/R-AKI)

AKI caused by renal I/R injury (I/R-AKI) emerges when blood circulation to kidneys suddenly decreases, with subsequent reperfusion generating an imbalance between oxygen and nutrient availability, harming endothelial cells and eventually progressing to kidney failure (Pan et al., 2021; Oh et al., 2023). I/R-AKI is implicated in cell death and tissue damage across various conditions, including AKI, stroke, and coronary occlusion. The I/R-AKI mechanism involves a complex series of interactions among inflammatory components, including oxidative stress, degradation of lipids through peroxidation, and reactive oxygen species generation, initiating an inflammatory sequence that results in cellular destruction and renal tissue damage (Shiva et al., 2020; Wu J. et al., 2022; Zhang B. et al., 2023). I/R-AKI frequently occurs in conditions such as septic shock, blood volume depletion, kidney transplantation, heart operations, and post-trauma stress. Recent studies (Junho et al., 2022; Wang S. et al., 2022; Yang C. et al., 2023) suggest that its pathogenesis is related to the mechanism of ROS. Inflammation, calcium overload, cell energy disturbance, and excessive cell apoptosis are closely related, and the main pathological changes are tubular epithelial necrosis and microvascular endothelial cell injury.

### 3.4 RM-induced AKI (RM-AKI)

Various clinical manifestations, encompassing disturbances in electrolytes, disorders of acid-base balance, abnormal coagulation, and impaired renal function, typify RM. The tissue damage in RM causes hazardous cellular components to enter the bloodstream, including myoglobin, creatine phosphokinase, and lactate dehydrogenase (Petejova and Martinek, 2014; Jiang et al., 2022; Williams et al., 2023). Among RM’s serious complications, AKI occurs in approximately 13%–50% of cases (Petejova and Martinek, 2014). The definite causing factors of RM-induced AKI remains incompletely known. The potential mechanisms might be related to myoglobin, a major renal toxin in RM and a key contributor to the disease, causing renal tubule obstruction, oxidative damage, and vasoconstriction, leading to AKI (Sun T. et al., 2022; Wang J. et al., 2022). In an acidic environment, myoglobin readily passes through glomerular filtration and accumulates in kidney tubules, and it is also easy to interact with Tamm Horsfall protein and precipitate in the renal tubules, thereby inducing lipid peroxidation and producing prostaglandins, resulting in renal arteriolar dysfunction and hypoperfusion.

### 3.5 Post-major surgeries induced AKI

AKI often occurs after major surgery. Mechanisms such as microembolism, neurohormonal activation, exogenous and endogenous toxins, hemodynamics, inflammatory factors, I/R injury, and OS may be involved. Those mechanisms might lead to renal changes: sustained vasoconstriction, overresponse to exogenous vasoconstrictors, and vascular endothelium, impairment of tubular cells, etc (Vives et al., 2019). During liver transplantation, blocked inferior vena cava and portal vein are prone to hypotension and intestinal congestion. Hypotension leads to renal hypoperfusion and I/R injury, and intestinal congestion promotes endotoxin production, which would result in AKI (Yuan et al., 2019). The pathogenesis of AKI after major surgery is complicated and multi-factors. The postoperative perfusion in renal tissue is reduced, and the compensatory blood volume in the kidney is increased, which dilates the entering arterioles and constricts the exiting arterioles, thereby maintaining glomerular filtration, leading to renal medullary ischemia and triggering AKI. In addition, anesthetics cause peripheral vasodilation and myocardial inhibition, impairing renal perfusion (Gameiro et al., 2018).

## 4 Applications of SMFH and phytochemicals of TCM against AKI

**FIGURE 2:**
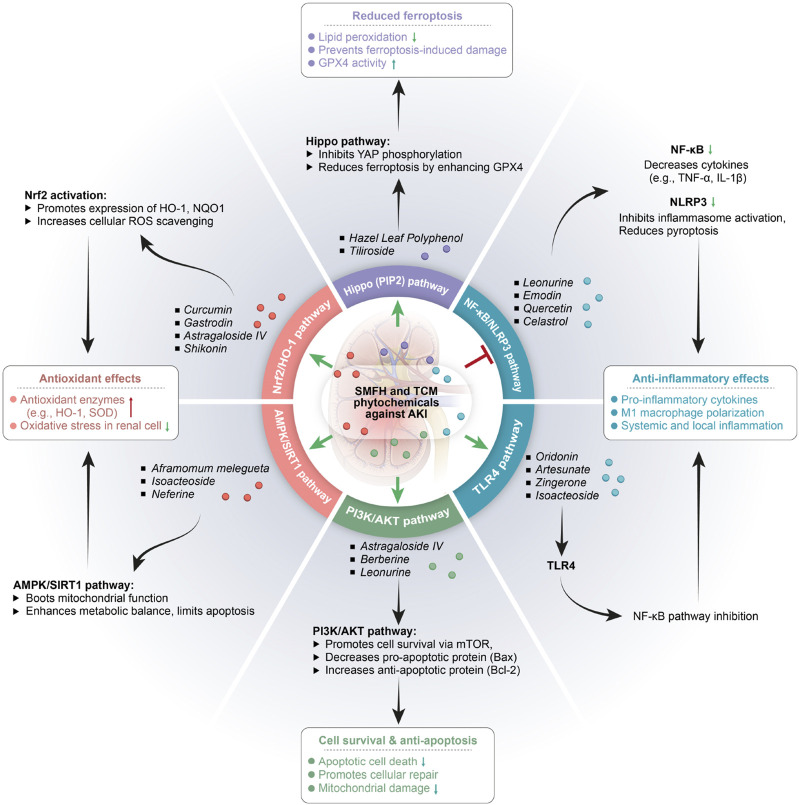
Substances of medicine food homology and phytochemicals of Traditional Chinese medicine in treatment of acute kidney injury. SMFH, substances of medicine food homology; TCM, traditional Chinese medicine; AKI, acute kidney injury; TNF-α, tumor necrosis factor-α; IL-1β, interleukin-1β; HO-1, Hemeoxygenase −1; NQO1, NAD(P)H quinone dehydrogenase 1; ROS, reactive oxygen species; NLRP3, NACHT, LRR, and PYD domains-containing protein 3; SOD, superoxide dismutase; NF-kB, nuclear factor kappa-Β.

TCM (including SMFH and TCM phytochemicals) possesses distinct therapeutic benefits in AKI prevention and treatments, encompassing its holistic philosophy, pattern identification methodology, and unique properties of its diverse components and targets (Figure 2). Prior researches have demonstrated that SMFH and TCM phytochemicals exert reno-protective effects by modulation of renal autophagy, inflammatory responses, and OS, etc., (Dong et al., 2021; Liu et al., 2022; Rui et al., 2022; Zhao et al., 2023). To illustrate comprehensively the nephroprotective effects of SMFH and TCM phytochemicals via multiple targets and pathways, we will elaborate from the following categories (Tables 1–4).

**TABLE 1:** Alkaloids of SMFH and TCM phytochemicals against AKI via various pathways and targets.

Phytochemicals	Original sources	Structures	AKI Models	Pathways and targets	References
Berberine	Rhizoma Coptidis	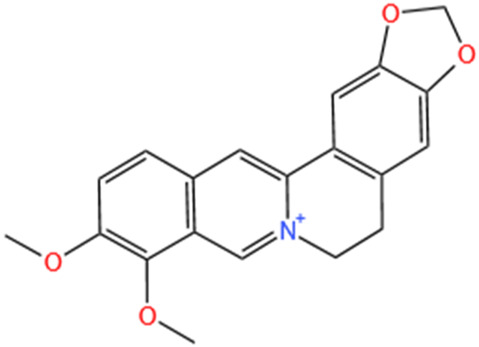	CI-AKI; Cis-induced AKI	Suppressing NLRP3 inflammasome and activation and modulating mitophagy; Regulating HDAC4-FoxO3a axis-induced autophagy; Activating Akt/Foxo3a/Nrf2 signalling pathway; Regulating mitophagy via PINK 1/Parkin pathway.	Qi et al. (2020), Wang et al. (2024b), Yang et al. (2024a), Zuo et al. (2024)
Leonurine	Leonurus Japonicus Houtt	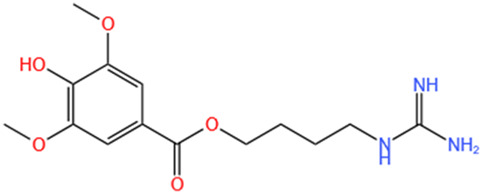	Cis-induced AKI; I/R-AKI; LPS-induced AKI	Inhibiting ER stress-associated ferroptosis via regulating ATF4/CHOP/ACSL4 pathway; Promoting Nrf2 Nuclear Translocation and Suppressing TLR4/ NF-κB Pathway; Suppressing NLRP3 infammasome, mitochondrial dysfunction, and endoplasmic reticulum stress; Suppressing ROS-mediated NF-κB signaling pathway.	Xu et al. (2014), Han et al. (2022), Zhang et al. (2022b), Cheng et al. (2024)
Tetramethylpyrazine	Ligusticum Chuanxiong Hort	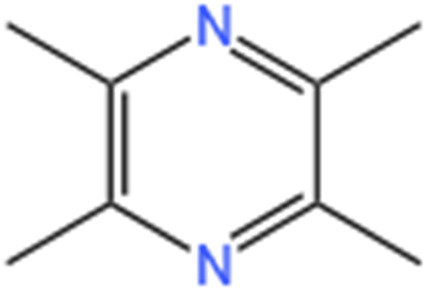	CI-AKI	Attenuating ferroptosis by inhibiting TFRC and intracellular ROS; Modulating mitophagy and suppressing mitochondrial fragmentation, CCL2/CCR2-mediated inflammation, and intestinal injury.	Gong et al. (2019), Zhu et al. (2024)
Emodin	Rhubarb	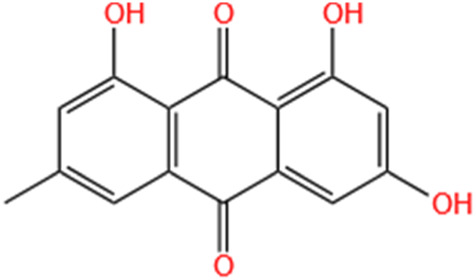	I/R-AKI; H/R-AKI	Suppressing p53-mediated cell apoptosis; Suppressing CAMKII/DRP1-mediated mitochondrial fission; Regulating apoptosis, ER stress, and ferroptosis.	Lin et al. (2021), Wang et al. (2022c), Lu et al. (2023)
Quercetin	Abelmoschus Manihot	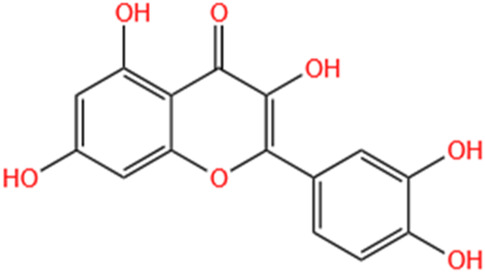	CI-AKI; COVID-19-induced AKI; Cis-induced AKI	Inhibiting HIF-1α/lncRNA NEAT1/HMGB1 pathway; Inhibiting inflammatory, cell apoptosis-related signaling pathways; Inhibiting Mincle/Syk/NF-κB signaling maintained macrophage inflammation.	Tan et al. (2020), Gu et al. (2021), Luo et al. (2022b)
Dihydroartemisinin	Artemisia Apiacea	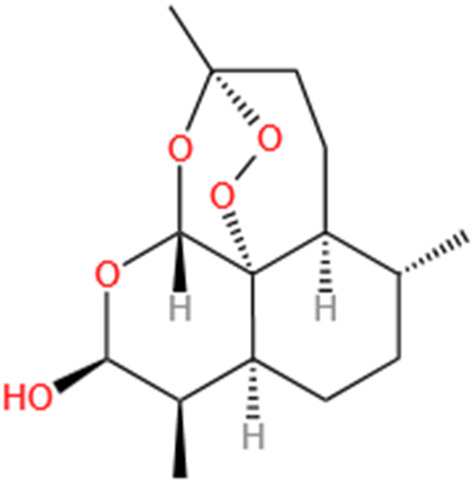	Cis-induced AKI; LPS-induced AKI	Inhibiting macrophagic Mincle-mediated necroptosis and inflammation; Inhibiting inflammation and oxidative stress; Upregulating occludin expression.	Cheng et al. (2018), Liu et al. (2019b), Lei et al. (2021)
Oridonin	Oridon	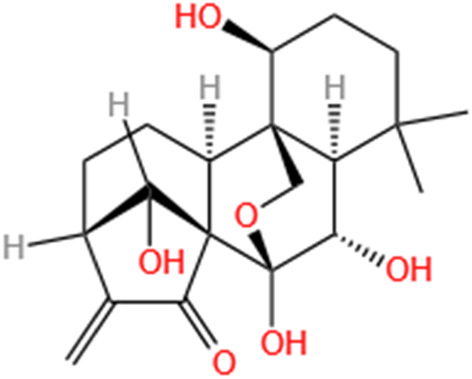	I/R-AKI; LPS-induced inflammatory BMDM cells	Suppressing macrophage involved inflammation; Inhibiting inflammatory response of macrophages via AKT-related pathways.	Yan et al. (2020), Tan et al. (2021)
Neferine	Lotus Plumule	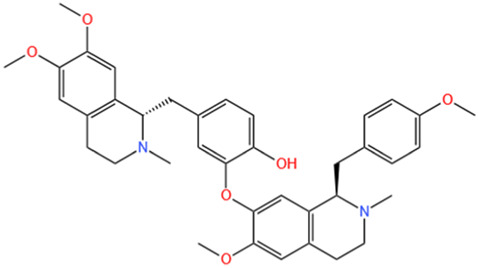	LPS-induced AKI; Cis-induced AKI	Regulating the PPAR-α/ NF-κB pathway; Regulating autophagy and apoptosis	Li et al. (2023a), Xiong et al. (2024)
Curcumin	Curcuma Longa	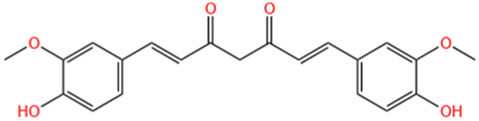	S-AKI; Cis-induced AKI	Regulating NF-κB and JAK2/STAT3 signaling pathway; Inhibiting Mincle-maintained M1 macrophage phenotype; Preventing alterations in mitochondrial bioenergetics, ultrastructure, redox balance, dynamic, and SIRT3 levels	Ortega-Domínguez et al. (2017), Tan et al. (2019), Zhu et al. (2020)
Celastrol	Tripterygium Wilfordii	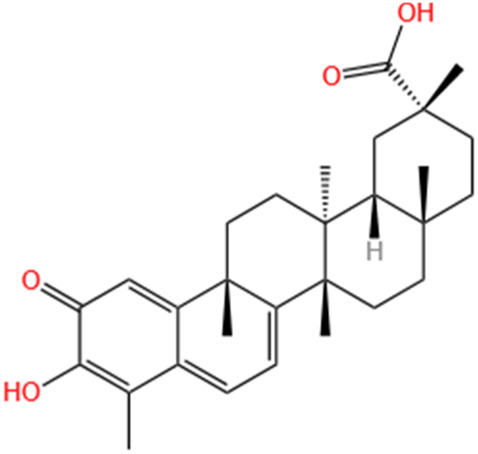	Cis-induced AKI	Inhibiting ferroptosis through Nrf2/GPX4 pathway; Inhibiting NF-κB and improving mitochondrial function	Yu et al. (2018), Pan et al. (2023)
Embelin	Embelia Ribes	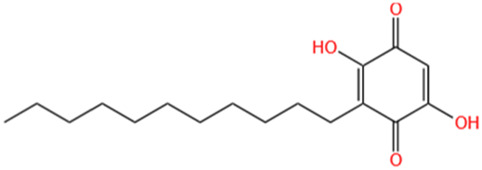	LPS-induced AKI	Inhibiting M1 macrophage activation and NF-κB signaling pathway.	Tang et al. (2023b)
Gastrodin	Gastrodia Elata Blume	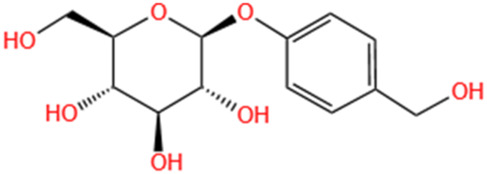	Cis-induced AKI	Inhibiting ferroptosis via the SIRT1/ FOXO3A/GPX4 signaling pathway.	Qiu et al. (2024)
Isorhamnetin	Ginkgo Biloba/Sea-Buckthorn	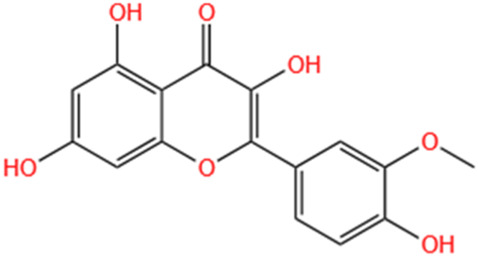	Cis-induced AKI	Activating SLPI-mediated anti-inflammatory effect in macrophage.	Jian et al. (2024)
Magnesium Lithospermate B	Salvia Miltiorrhiza Bunge	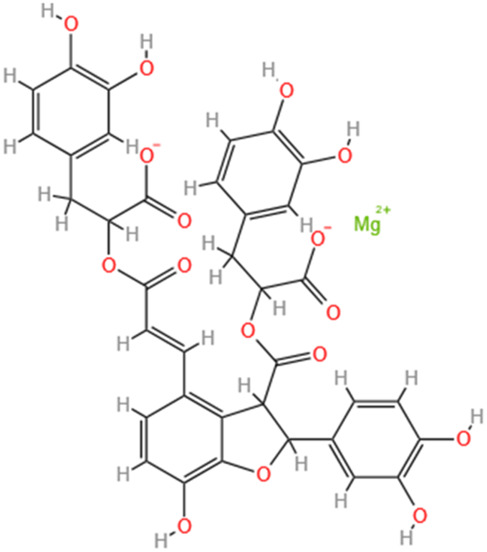	Cis-induced AKI	Alleviating Mitochondrial Dysfunction.	Shen et al.(2022)
Shikonin	Radices Lithospermi	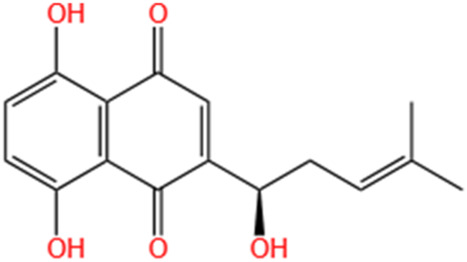	CLP-induced AKI; LPS-stimulated HK-2 cells and TECs	Modulating NOX4/PTEN/AKT pathway.	Peng et al. (2022)
Liquiritigenin	Glycyrrhiza Uralensis Fisch	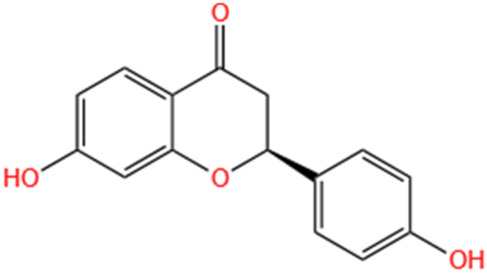	Cis-induced AKI	Activating NRF2/SIRT3-Mediated Improvement of Mitochondrial Function.	Zhou et al. (2022b)
Oroxylin A	Semen Oroxyli	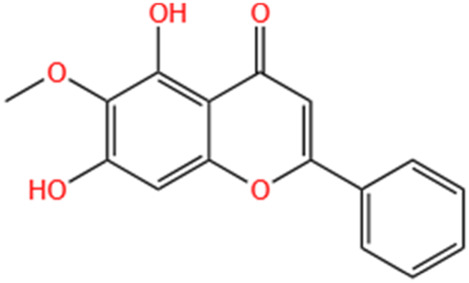	Cis-induced AKI	Maintaining mitochondrial homeostasis via inducing PPARα-BNIP3 signaling pathway.	Yao et al. (2022b)
Arbutin	Chinese Yam	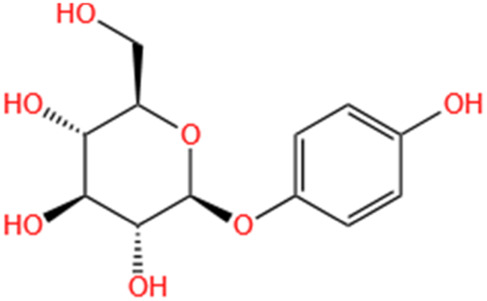	LPS-induced AKI	Inhibiting inflammation and apoptosis via the PI3K/Akt/Nrf2 pathway.	Zhang et al. (2021b)
Puerarin	Radix Puerariae	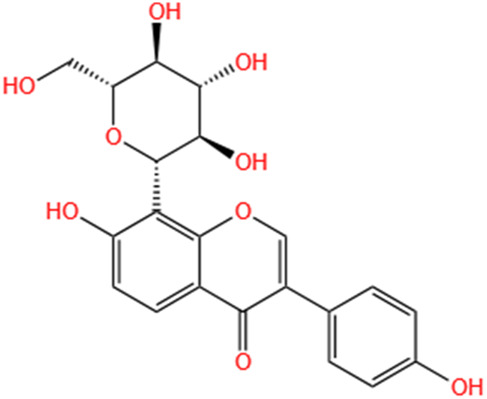	Cis-induced AKI	Upregulating microRNA-31-related signaling pathway.	Wu et al. (2020)
Isoorientin	Gentiana/Patrinia	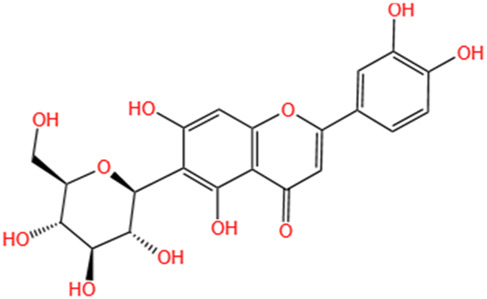	Cis-induced AKI	Inhibiting oxidative stress and apoptosis via activating the SIRT1/SIRT6/Nrf-2 pathway.	Fan et al. (2020)

SMFH: substances of medicine food homology; TCM: traditional Chinese medicine; AKI: acute kidney injury; CI-AKI: contrast-induced acute kidney injury; Cis-induced AKI: cisplatin -induced acute kidney injury;NLRP3: nucleotide-binding oligomerization domain-like pyrin domain-containing protein 3; ER: endoplasmic reticulum; I/R-AKI:ischemia/reperfusion-induced acute kidney injury; LPS: lipopolysaccharide;TFRC: transferrin receptor;ROS: reactive oxygen species; H/R-AKI: hypoxia/reoxygenation-induced acute kidney injury; COVID-19-induced AKI: coronavirus infection disease 2019-induced acute kidney injury; BMDM: bone marrow-derived macrophages; SAKI: sepsis acute kidney injury; SLPI: secretory leukocyte peptidase inhibitor; CLP-induced AKI: cecal ligation and perforation-induced acute kidney injury; HK-2 cells:human kidney-2 cells; TECs: tubular epithelial cells;

**TABLE 2:** Saponins of SMFH and TCM phytochemicals against AKI via various pathways and targets.

Phytochemicals	Original sources	Structures	AKI Models	Pathways and targets	References
Astragaloside IV	Astragalus Membranaceus	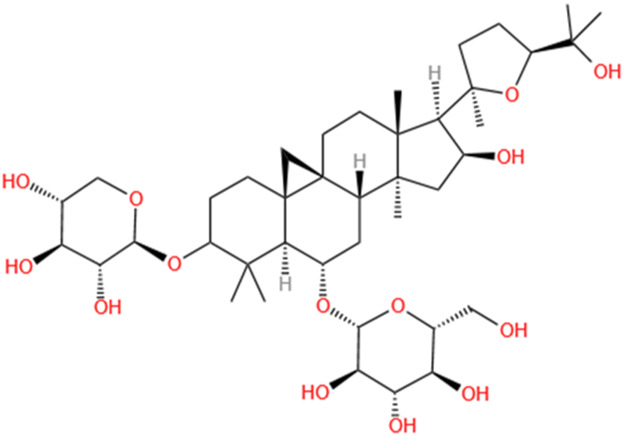	S-AKI; I/R-AKI; CI-AKI	Activating Gpr97-TPL2 signaling pathway; Inhibiting endothelial ferroptosis; Activating the PI3K/AKT pathway; Inhibiting oxidative stress and apoptosis pathways.	Gui et al. (2013) Tang et al. (202)2, Guo et al. (2023), Xu et al. (2024)
Rehmaionoside C	Rehmannia Glutinosa	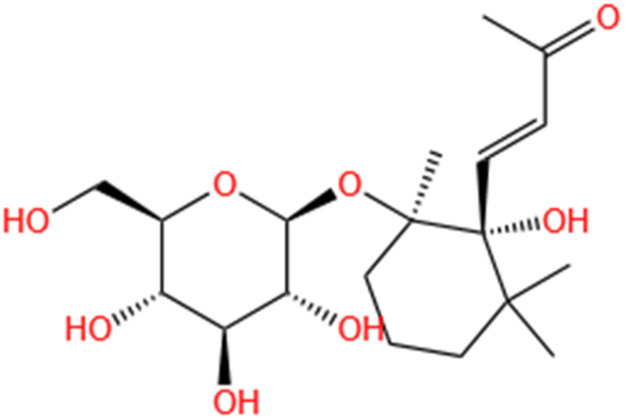	LPS-induced AKI	Regulating ER-TLR4-IL-1β pathway and ERα and ERβ receptors.	Liu et al. (2024b)
Ginsenoside Rg1	Panax Ginseng	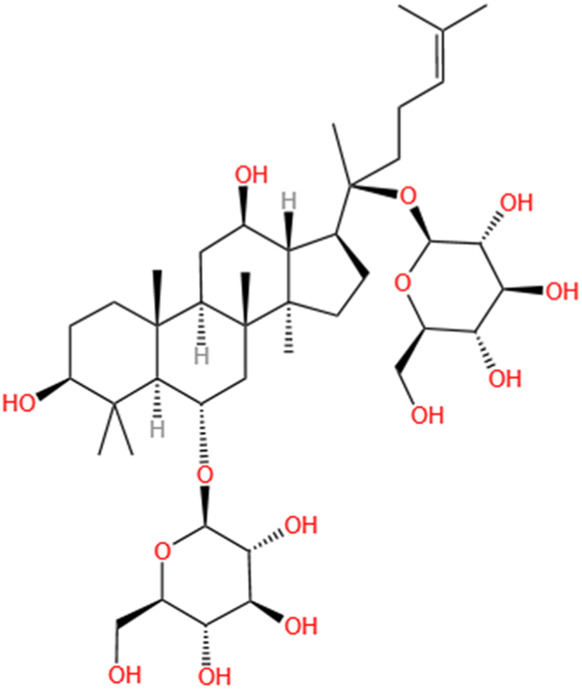	LPS-induced AKI	Regulating the SIRT1/NF-κB signaling pathway.	Hu et al. (2024)
Polydatin	Polygonum Cuspidatum	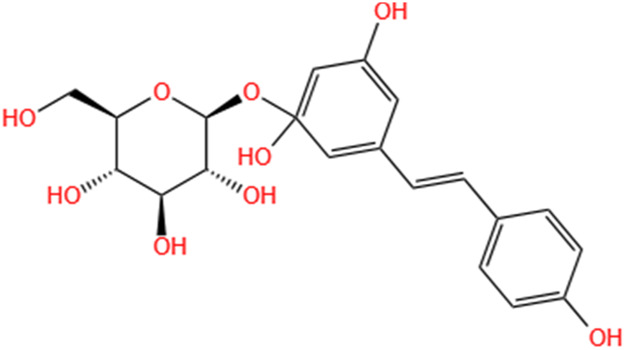	S-AKI; Cis-induced AKI; LPS-induced AKI	Inhibiting NF-κB-mediated inflammation and pyroptosis; Inhibiting ferroptosis via maintenance of the system Xc- - GSH-GPx4 axis and iron metabolism; Inhibiting inflammatory and oxidative responses.	Gu et al. (2019), Zhou et al. (2022a), Yang et al. (2024b)
Tiliroside	Tribulus Terrestris	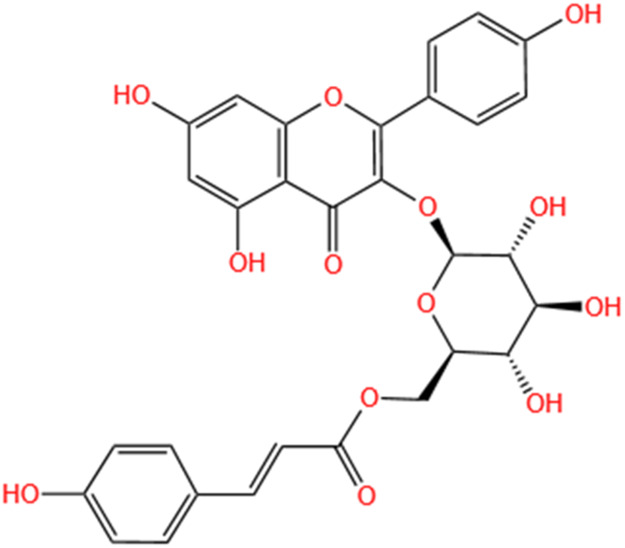	Cis- and I/R-induced AKI mouse and HK2 cells models; LPS-induced AKI	Inhibiting ferroptosis through the disruption of NRF2-KEAP1 interaction; Activating autophagy flux via intrarenal renin–angiotensin system.	Yi et al. (2023), Cai et al. (2024)
Paeoniflorin	Paeonia Lactiflora Pall	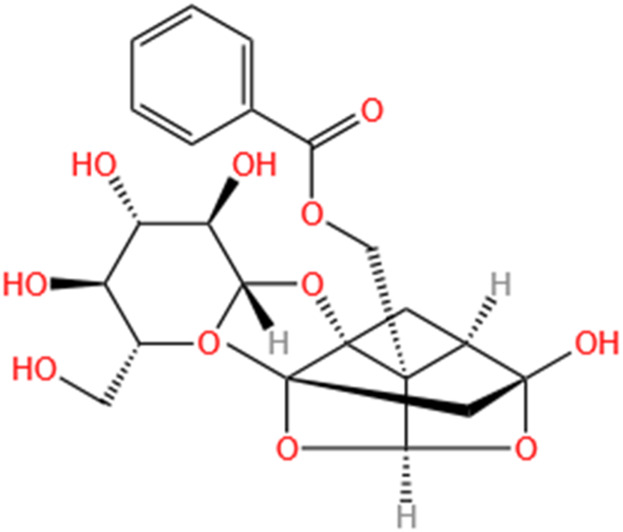	Cis-induced AKI; I/R-AKI; H/R-AKI in HK-2 cells	Promoting Hsp90AA1-Akt protein-protein interaction; Inhibiting SlC7A11-mediated ferroptosis; Inhibiting apoptosis and repressing oxidative damage via Keap1/ Nrf2/HO-1 pathway.	Ma et al. (2023), Xing et al. (2023), Zhang et al. (2023b)
Hyperoside	Abelmoschus Manihot (L.) Medic	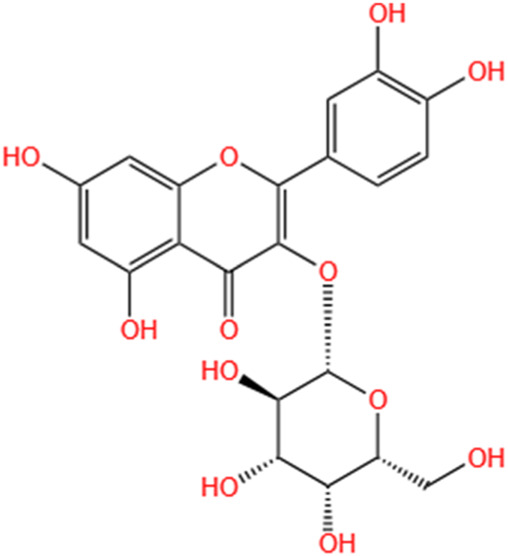	Cis-induced AKI; I/R-AKI	Regulating the expression and function of Oat1; Modulating mitochondrial fission, oxidative stress, and apoptosis.	Wu et al. (2019), Yuan et al. (2023)
Monotropein	Morinda Officinalis	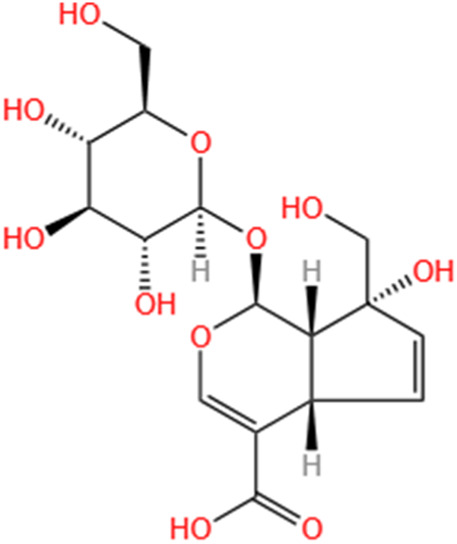	Cis-induced AKI	Inhibiting oxidative damage, inflammation and apoptosis.	Zhang et al. (2020a)
Forsythiaside A	Forsythia	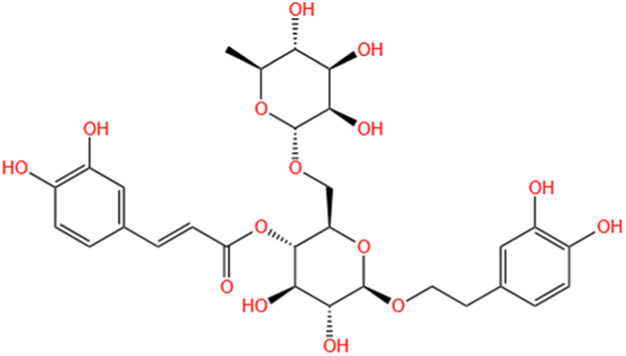	S-AKI	Anti-infammation and antiapoptotic effects by regulating PERK signaling dependent ER stress responses.	Chen et al. (2023)
Salidroside	Rhodiola Rosea	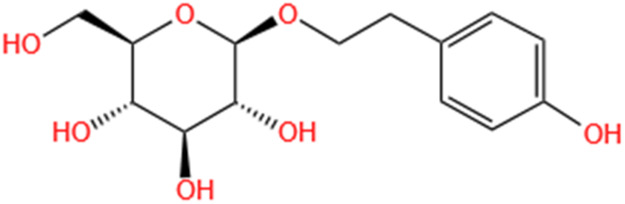	I/R-AKI	Inhibiting ferroptosis by the PI3K/AKT signaling pathway.	Tang et al. (2023c)
Notoginsenoside Fc	Panax Notoginseng	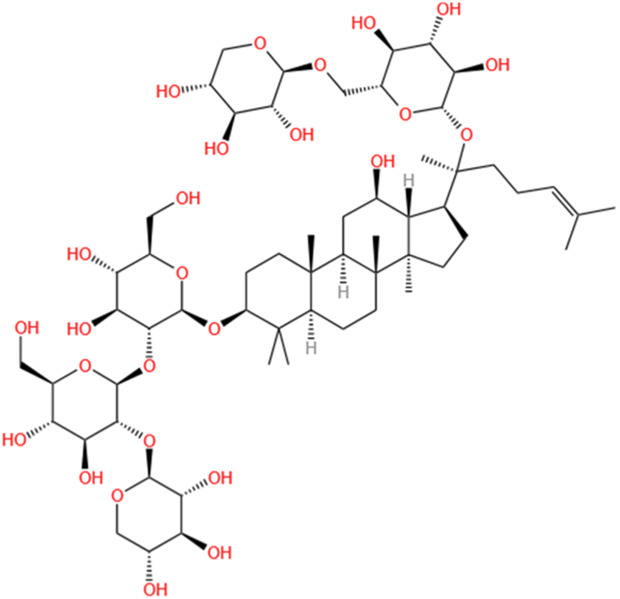	APAP-induced AKI	Regulating of SIRT3/SOD2 pathway.	Wei et al. (2022)
Capilliposide A	Lysimachia Capillipes Hemsl.	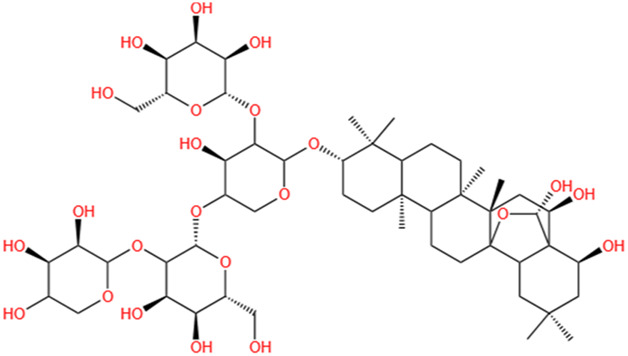	Cis-induced AKI mouse and HK2 cells models	Regulating endogenous metabolites , ameliorating apoptosis and oxidative stress by reducing ER stress.	Fang et al. (2024)
Gypenoside XVII	Gynostemma Pentaphyllum	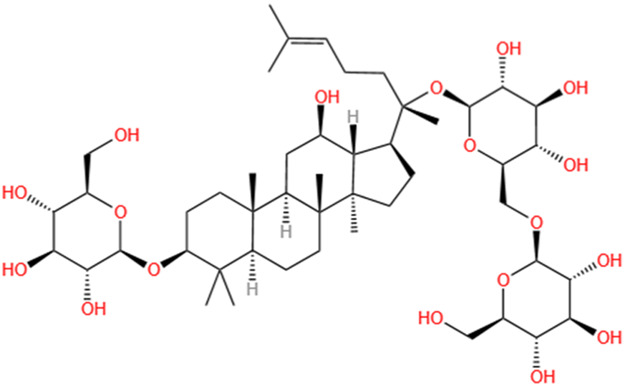	I/R-AKI	Inhibiting ER stress and NLRP3 inflammasome-triggered pyroptosis.	Wang et al. (2024a)
Icariin	Epimedium Brevicornu Maxim	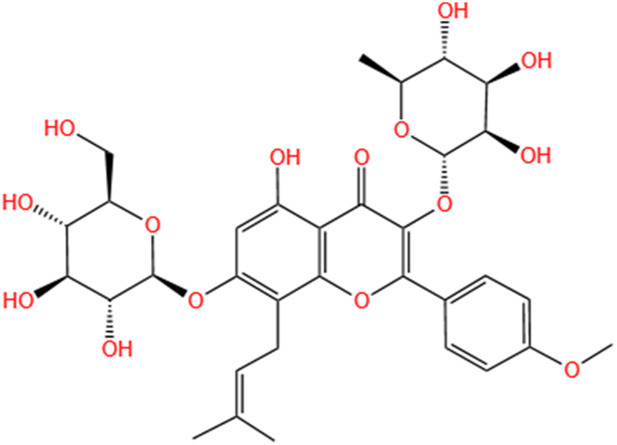	FA-induced AKI	Inhibiting complement and coagulation cascade signals.	Zhang et al. (2024a)
Hederagenin	Astragalus	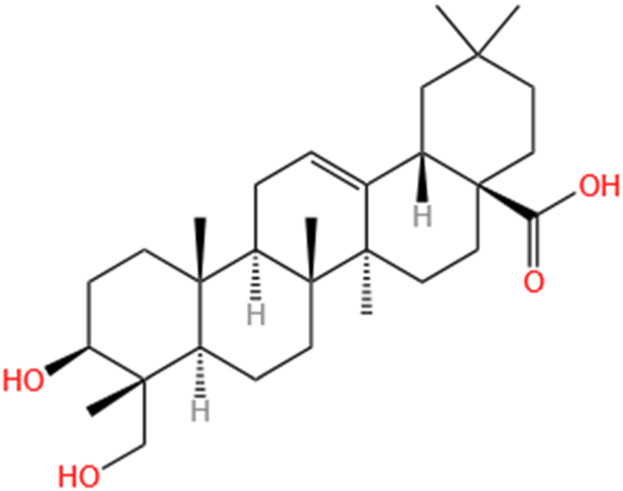	Cis-induced AKI	Inhibiting long non-coding RNA A330074k22Rik/Axin2/β-catenin signalling pathway.	Xie et al. (2022)
Trilobatin	Lithocarpus Polystachyus Rehd	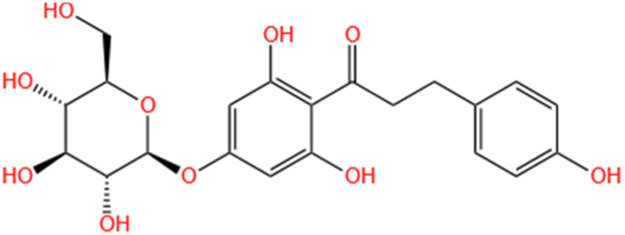	Cis-induced AKI	Regulating the AKT/MAPK signaling pathway and apoptosis.	Duan et al. (2022)
Isoacteoside	Monochasma Savatieri Franch. ex Maxim	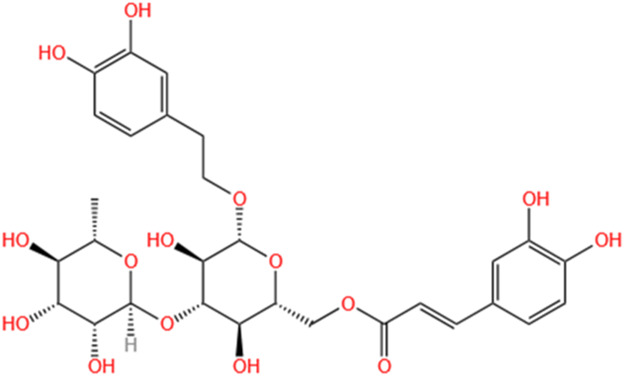	SAP-induced AKI	Regulating TLR4/NF-κB signaling pathway.	Wang et al. (2021a)
Oleuropein	Ilex Pubescens Hook. et Arn. var. Kwangsiensis Hand.-Mazz.	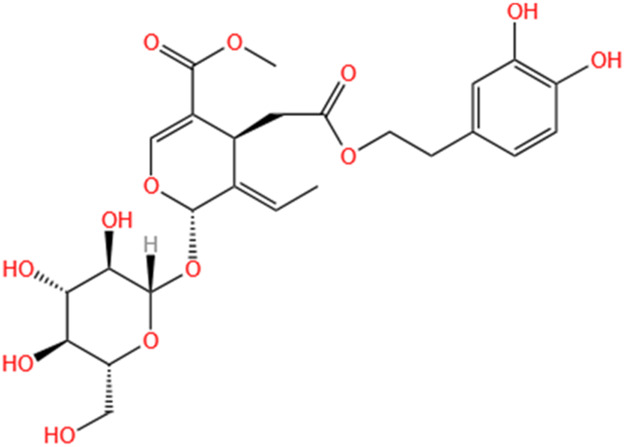	LPS-induced AKI	Regulating TLR4-MyD88-NF-κB/MAPK axis.	Cui et al. (2021)
Loganin	Corni Fructus	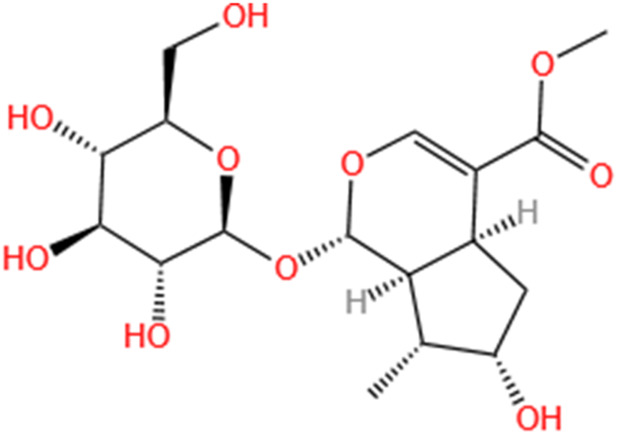	Cis-induced AKI	Inhibiting ERK 1/2 activation.	Kim et al. (2021)
Nodakenin	Umbelliferae	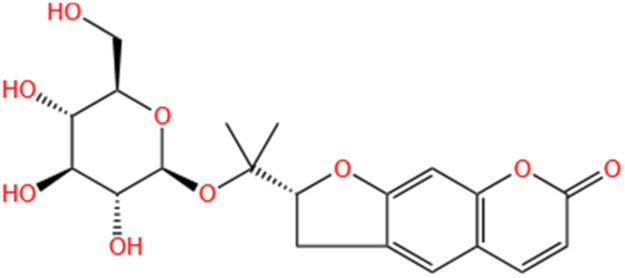	I/R-AKI	Inhibiting ROS induced NLRP3 inflammasome activation.	Liao et al. (2021)
Arginyl-fructosyl-glucose	Red Ginseng	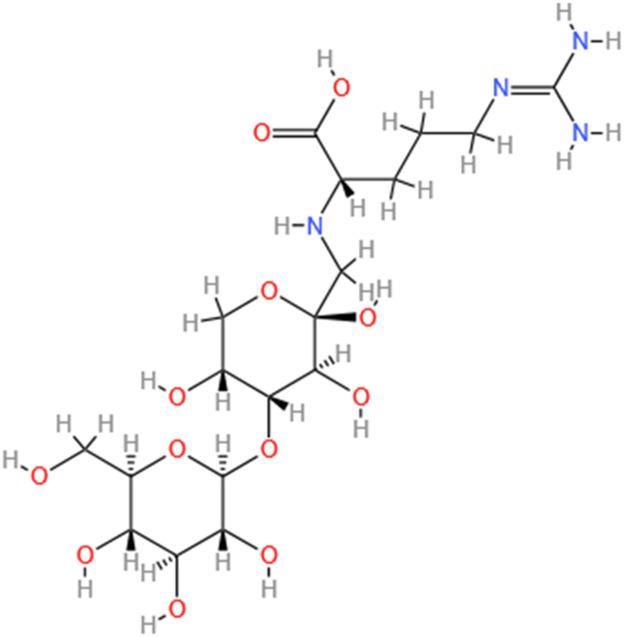	Cis-induced AKI	Regulating 3 NF-κB and PI3K/Akt Signaling Pathways	Li et al. (2019c)
Eleutheroside B	Acanthopanax Senticosus	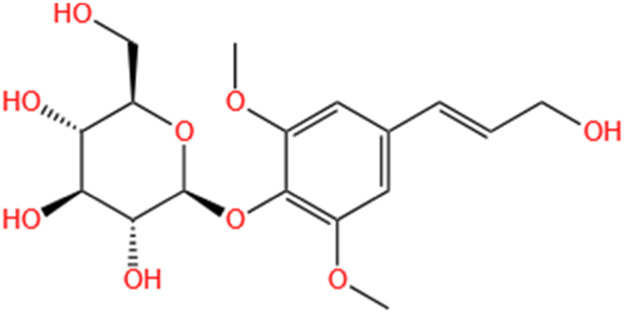	Cis-induced AKI mouse and HK2 cells models	Activating IGF pathway.	Zang et al. (2019)
Esculentoside A	Phytolaca Esculenta	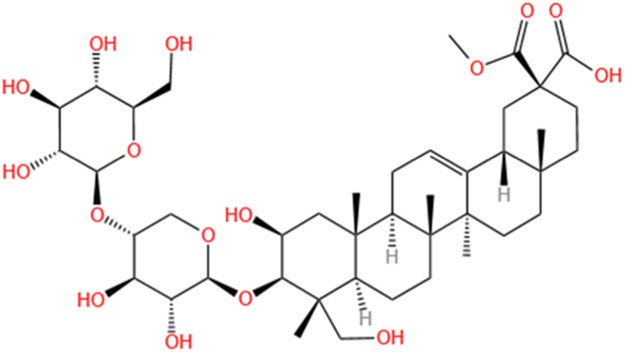	LPS-induced AKI	Activating PPAR-γ.	Chen et al. (2017)

SMFH: substances of medicine food homology; TCM: traditional Chinese medicine; AKI: acute kidney injury; S-AKI: sepsis associated acute kidney injury; I/R-AKI:ischemia/reperfusion-induced acute kidney injury; CI-AKI: contrast-induced acute kidney injury; LPS: lipopolysaccharide; ER: estrogen receptor; Cis-induced AKI: cisplatin -induced acute kidney injury; H/R-AKI: hypoxia/reoxygenation-induced acute kidney injury; ER stress: endoplasmic reticulum stress; APAP-induced AKI: acetaminophen-induced acute kidney injury; NLRP3: nucleotide-binding oligomerization domain-like pyrin domain-containing protein 3; FA-induced AKI: folic acid-induced acute kidney injury; SAP-induced AKI: Severe acute pancreatitis-induced acute kidney injury; ROS: reactive oxygen species.

**TABLE 3:** Flavonoids, Polyphenols,and Lipoid of SMFH and TCM phytochemicals against AKI via various pathways and targets.

Categories	Phytochemicals	Original sources	Structures	AKI Models	Pathways and targets	References
Flavonoids	Tanshinone IIA	Salvia Miltiorrhiza	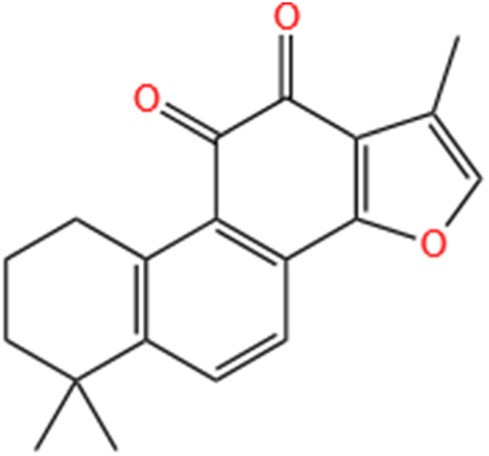	Cis-induced AKI; I/R-AKI; FA-induced AKI	Regulating PXR/NF-κB signaling; Modulating mitochondrial function through PI3K/Akt/Bad pathway; Alleviates renal tubular epithelial cells damage, ameliorating inflammatory response, and preventing long-term kidney fibrosis; Targeting GSK3β.	Jiang et al. (2016b), Tai et al. (2021a), Tai et al. (2021b), Dou et al. (2022), Tai et al. (2022)
	Shionone	Rhizome of Aster tataricus L. f.	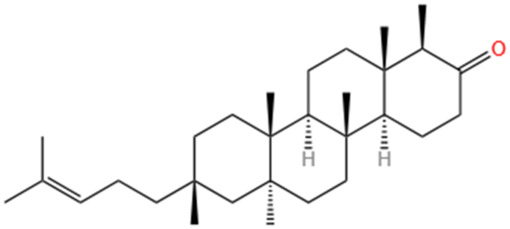	CLP-induced AKI mouse and LPS-stimulated RAW264.7 cells models	Promoting M2 macrophage polarization through regulating the ECM1/STAT5 pathway.	Zhang et al. (2021a)
	5-O-methyldihydroquercetin	Spina Gleditsiae	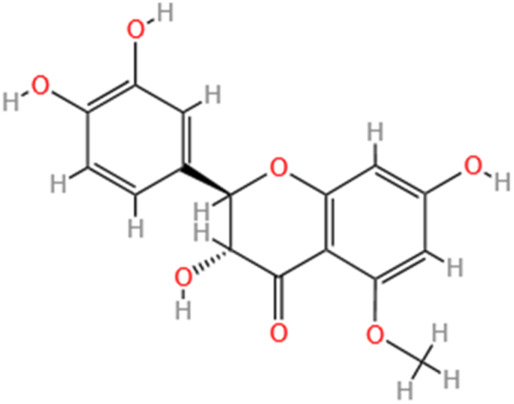	LPS-induced AKI	Inhibiting inflammation and oxidative stress via the TLR4/MyD88/TRIF/ NLRP3 signaling pathway.	Zeng et al. (2020a)
	Zingerone	Ginger	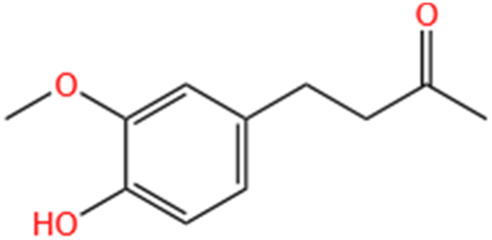	LPS-induced AKI	Suppressing TLR4/NF-κB signaling pathway.	Song et al. (2016)
Polyphenols	Salvianolic Acid A	Salvia Miltiorrhiza	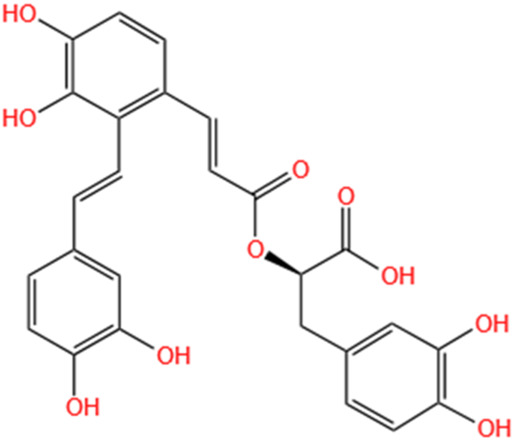	Gentamicin-induced AKI; LPS-induced AKI; I/R-AKI	Regulating the MAPKs and TGF-β1/smads signaling pathways; Suppressing inflammatory response; Protecting against peritubular capillary endothelium damages.	Zhang et al. (2018b), Zeng et al. (2020b), Diao et al. (2023)
	Salvianolic Acid C	Salvia Miltiorrhiza	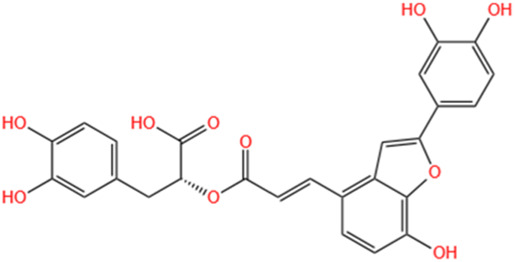	Cis-induced AKI	Attenuating inflammation, oxidative stress and apoptotic effects and activating the CaMKK–AMPK–Sirt1-associated signaling pathway.	Chien et al. (2021)
	Salvianolic Acid B	Salvia Miltiorrhiza	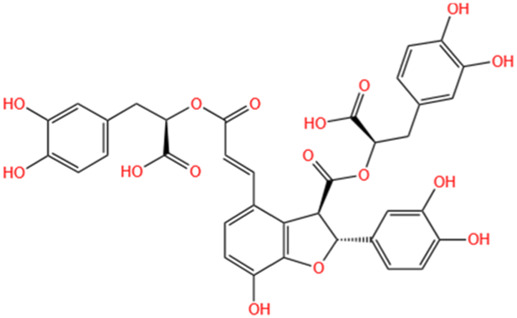	I/R-AKI	Inhibit caspase-1/GSDMD-mediated pyroptosis by activating Nrf2/NLRP3 signaling pathway.	Pang et al. (2020)
	Salvianolate	Salvia Miltiorrhiza	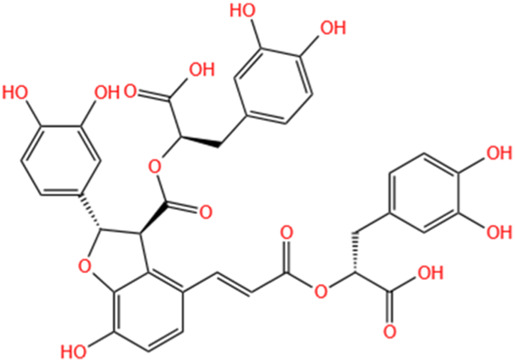	I/R-AKI	Exerting anti-apoptotic effects via activation of the Keap1-Nrf2-ARE signaling pathway.	Sun et al. (2022a)
	Honokiol	Magnolia Officinalis	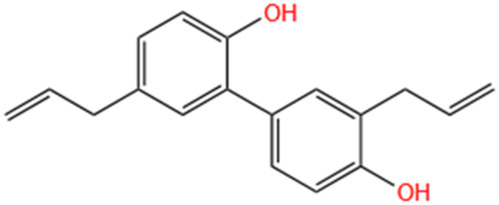	S-AKI	Inhibiting oxidative stress and inflammation.	Xia et al. (2019a)
Lipoid	Artesunate	Artemisia Apiacea	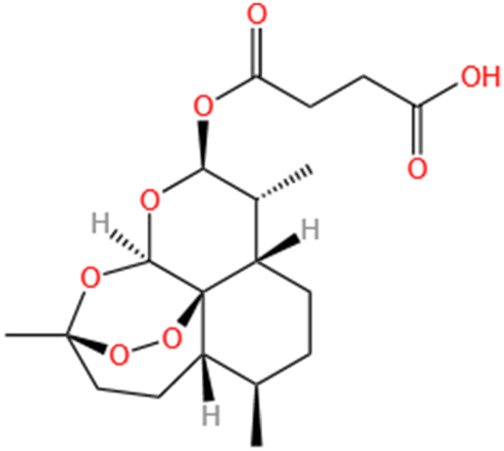	Cis-induced AKI	Inhibiting macrophagic Mincle-mediated necroptosis and inflammation.	Lei et al. (2021)
	Limonin	Fructus Evodiae	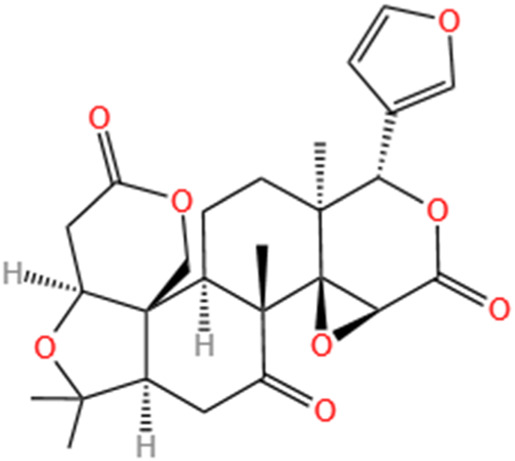	I/R-AKI	Activating ERK signaling pathway.	Zhou et al. (2023)
	Parthenolide	Tanacetum Vulgare L.	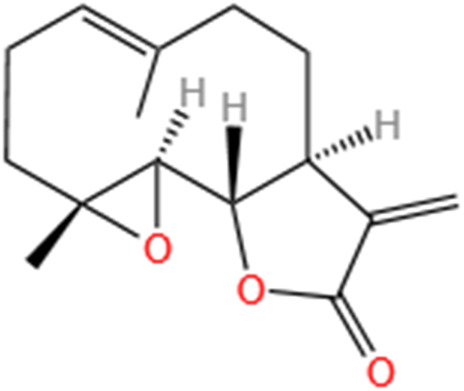	CLP-induced AKI mouse and LPS-stimulated rat glomerular mesangial cells models	Suppressing NF-κB signaling.	Shou et al. (2023)
	Alisol B 23-Acetate	Alismatis Rhizoma	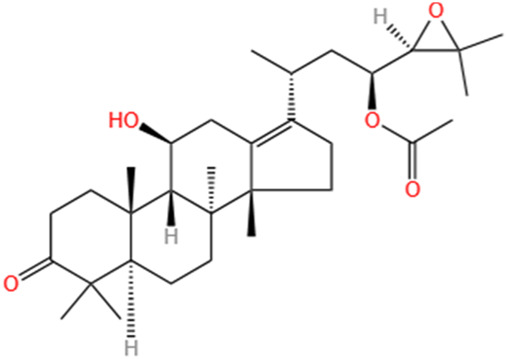	I/R-AKI	Activating renal FXR to exert renoprotection.	Luan et al. (2021)
	Wedelolactone	Eclipta Prostrata	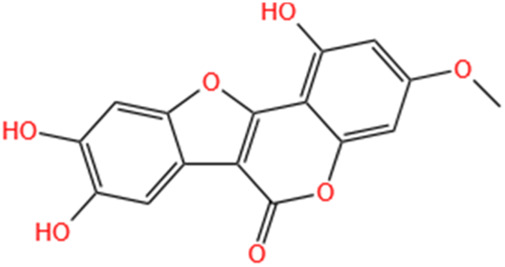	DOX-induced AKI	Inhibiting IκK/IκB/NF-κB pathway.	Zhu et al. (2019)

SMFH: substances of medicine food homology; TCM: traditional Chinese medicine; AKI: acute kidney injury; Cis-induced AKI: cisplatin -induced acute kidney injury; I/R-AKI:ischemia/reperfusion-induced acute kidney injury; FA-induced AKI: folic acid-induced acute kidney injury; CLP: cecum ligation and puncture; LPS: lipopolysaccharide; S-AKI: sepsis associated acute kidney injury; DOX:doxorubicin; EMT: Epithelial mesenchymal transition.

**TABLE 4:** Other SMFH and TCM phytochemicals against AKI via various pathways and targets.

Phytochemicals	Original sources	AKI models	Pathways and targets	References
Cordyceps sinensis extract	Cordyceps sinensis	FA-induced AKI	Inhibiting perforin expression in NK cells via the STING/IRF3 pathway	Li et al. (2024)
Polygonum cuspidatum Sieb. et Zucc. Extracts	Polygonum Cuspidatu m	S-AKI	Inhibiting NF-κB-mediated inflammation and pyroptosis	Yang et al. (2024b)
Leontopodium leontopodioides extracts	Leontopodium leontopodioides (Willd.) Beauv	LPS-induced AKI	Inhibiting NF-κB/NLRP3 pathway	Bai et al. (2023)
Hederasaponin C	Pulsatilla chinensis (Bunge) Regel	LPS-induced AKI	Targeting TLR4 and regulating the PIP2/NF-κB/NLRP3 signaling pathway	Han et al. (2023)
*Achyranthes aspera* extract	*Achyranthes aspera*	Cis-induced AKI	Alleviating oxidative stress, inflammation, and PANoptosis	Lin et al. (2024)
Hazel Leaf Polyphenol Extract	Hazelnuts	Cis-induced AKI	Reducing ferroptosis through Inhibiting Hippo Signaling pathway	Sun et al. (2024)
*Agathis robusta* Bark Extract	*Agathis Robusta*	I/R-AKI	Downregulating of HSP90 and P53	Mohamed et al. (2022)
Aframomum melegueta seeds extract	Aframomum melegueta	DIC-induced AKI	Activating Nrf2/HO1 and AMPK/SIRT1, and inhibiting NF-ҡB/STAT3 signaling pathways	Abdou et al. (2021)
Ethanol Extract of Illicium henryi	Illicium henryi Diels	LPS-induced AKI	Regulating inflammation and oxidative stress	Islam et al. (2019)
Ferulic acid	Angelica sinensis/Cimicifuga heracleifolia/Lignsticum chuangxiong	LPS-induced AKI	Suppressing inflammatory events and upregulating antioxidant defenses	Mir et al. (2018)
Tribulus terrestris L extract	Tribulus terrestris	I/R-AKI	Decreasing kidney functional disturbance, oxidative stress, and cellular damages	Najafi et al. (2014)

SMFH, substances of medicine food homology; TCM, traditional Chinese medicine; AKI, acute kidney injury; FA-induced AKI: folic acid-induced acute kidney injury; NK, cells: Natural killer cells; S-AKI, sepsis associated acute kidney injury; LPS, lipopolysaccharide; Cis-induced AKI, Cisplatin-induced acute kidney injury; DIC-induced AKI, Diclofenac-induced acute kidney injury.

### 4.1 Alkaloids

#### 4.1.1 Berberine

Berberine (BBR), extracted from the rhizomacoptidis, possesses multi-pharmacological effects, including antioxidative effects, reduction of mitochondrial injury, anti-inflammatory actions, autophagy regulation, and cell death (Hashemzaei and Rezaee, 2021; Rui et al., 2022). Mitophagy has been recognized as a vital mechanism for eliminating damaged mitochondria and facilitating cellular repair. Yang et al. (2024a) indicated BBR effectively mitigated contrast-induced AKI (CI-AKI) mice by inhibiting NLRP3 inflammasome activation and promoting mitophagy. Qi et al. (2020) elucidated that the mitophagy induced by BBR was mediated through PINK1/Parkin pathway activation in mice and renal tubular epithelial cells (RTECs). In the context of CI-AKI, the activation of mitophagy by BBR may counteract damaging effects via the reduction of ROS accumulation. Furthermore, BBR could diminish metabolic disturbances, inflammation, and apoptosis induced by cisplatin-mediated methylation (Shen et al., 2020). Significantly, BBR has emerged as a widespread inhibitor of HDACs and is capable of down-regulating HDAC4, thereby enhancing epigenetic modifications through increased histone acetylation (Kandasamy et al., 2023). Zuo et al. (2024) evaluates the renal protective effects of BBR and its potential mechanisms involving HDAC4. BBR exhibited marked protective outcomes against CI-AKI, preventing apoptosis by enhancing Bcl-2 protein expression while diminishing Bax levels. Their research also demonstrated that the ioversol contrast medium increased HDAC4 expression. Wang et al. (Wang W. et al., 2024) found BBR effectively counteracted ioversol-triggered apoptosis and ferroptosis. BBR’s protective mechanisms operated through regulation of Akt/Foxo3a/Nrf2 signaling cascade, resulting in CI-AKI improvement.

#### 4.1.2 Leonurine

Leonurine (LEO) is a distinctive alkaloid that demonstrates a variety of bioactivities, encompassing antioxidant, anti-apoptotic, anti-inflammatory properties, and improve microcirculation (Liu et al., 2012; Zhu et al., 2018). In a gouty arthritis model, LEO has been shown to suppress NLRP3 activation and diminish IL-1β and TNF-α generation (Liu Y. et al., 2018). Xu et al. (2014) revealed LEO exerted significant nephroprotective effects in an LPS mice model. The inhibition of proinflammatory cytokine production, maintenance of redox balance, and suppression of ROS-mediated NF-κB signaling activation may mediate the nephroprotective properties of LEO. Zhang Q. et al. (2022) indicated that LEO serves a protective function in CI-AKI and could function as a viable therapeutic approach. The kidney-protective properties of LEO against cell death appear to be mediated through diminished NLRP3 and inflammatory mediators, improving mitochondrial function, and attenuating endoplasmic reticulum (ER) stress. In renal disease treatment, LEO has been demonstrated to ameliorate AKI induced by LPS, mitigate adriamycin-related podocyte injury, and reduce the nephrotoxic effects of cisplatin (Hu et al., 2022; Zhang Q. et al., 2022). Chen P. et al. (2019) reported that LEO decreases OS in aging mice by activating Nrf2 pathway. Han et al. (2022) showed LEO pretreatment could alleviate ischemic AKI by promoting Nrf2 nuclear translocation, which counteracts OS injury and inhibits TLR4/NF-κB pathway that mediates inflammatory expression. A recent study (Wu et al., 2023) discovered LEO’s *in vitro* anti-ferroptotic capabilities, achieved partly via p62/Nrf2/HO-1 pathway activation. LEO diminishes AKI and preserves renal function by controlling ferroptosis and reducing endoplasmic reticulum stress. Significantly, ER stress demonstrates close connections with erastin-triggered ferroptosis. Cheng et al. (2024) unveiled fresh perspectives regarding LEO’s protective mechanisms against AKI, emphasizing its capacity to suppress ER stress-associated ferroptosis through ATF4/CHOP/ACSL4 pathway regulation.

#### 4.1.3 Tetramethylpyrazine

Tetramethylpyrazine (TMP) is identified as a bioactive constituent within Chuanxiong, exhibiting nephroprotective properties attributed to its antioxidant capabilities (Li and Gong, 2022). The potential mechanisms through which TMP acts against AKI have been reviewed by Li et al. (Li and Gong, 2022). The findings suggest that the mechanisms involved in TMP’s protective effects against AKI primarily revolve around the mitigation of OS injury, suppression of inflammation, prevention of apoptosis in intrinsic renal cells, and regulation of autophagy. It has been demonstrated TMP can reduce renal damage in a CI-AKI animal model via multiple pathways, with the modulation of tubular epithelial cell death paramount. In the CI-AKI mouse model, significant attenuation of renal tubular cell apoptosis by TMP was observed, potentially mediated by suppressing the p38 MAPK and FoxO1 pathways (Gong et al., 2013). Studies (Gong et al., 2015; Gong et al., 2016) have underscored the potential of TMP as an innovative therapeutic option in CI-AKI prevention by suppressing the p38 MAPK and FoxO1 pathways, additionally protecting renal tubular cells against arsenite-induced nephrotoxicity by averting mitochondrial dysfunction and regulating autophagy. Gong et al. (2019) demonstrated that TMP suppresses CCL2/CCR2 pathway activation in CI-AKI, alleviates renal OS and abnormal mitochondrial dynamics, and modulates mitochondrial autophagy within renal tubular cells. Zhu et al. (2024) explored involvement of ferroptosis in RTECs concerning the reno-protective effects of TMP against CI-AKI and the molecular mechanisms governing TMP’s regulation of ferroptosis. The results indicated that tubular cell injury was associated with ferroptosis, evidenced by elevated Fe^2+^ levels, lipid peroxidation, and reduced GPX4 levels. Furthermore, TMP markedly inhibits renal dysfunction, lowers AKI biomarkers, prevents ROS production, diminishes renal Fe^2+^ accumulation, and enhances GPX4 expression. Findings from siRNA silencing and plasmid amplification of transferrin receptor (TFRC) suggested TFRC is crucial for TMP’s ability to mitigate ferroptosis and decrease LDH release, Fe^2+^ buildup, and intracellular ROS.

#### 4.1.4 Emodin

Emodin is recognized as an anthraquinone derivative derived from the rhizome of Rheum palmatum L, serving as the principal active monomer in Da Huang. Recent evidence (Ma et al., 2018; Xia et al., 2019b) has demonstrated that emodin has antibacterial, anti-inflammatory, antioxidant, immunosuppressive, and anti-renal fibrosis effects. Lu et al. (2023) integrated network pharmacology with experimental validation to assess the preventive efficacy and underlying mechanisms of emodin in the context of AKI. These effects are likely linked to its anti-apoptotic responses and enhancement of angiogenesis through the regulation of the p53/Caspase-9/Caspase-3, p53/Bcl-2, and HIF-1α/VEGF signaling pathways. Li et al. (2015) demonstrated emodin effectively protects NRK-52E cells from LPS-induced injury by downregulating expression of TLR2, NF-κB, and inflammatory cytokines. Furthermore, emodin demonstrated suppressive effects on LPS-triggered TLR2 and NF-κB expression within NRK-52E cells. Upon administration of emodin, a concentration-dependent reduction was noted in both mRNA and protein expressions of NF-κB, TNF-α, IL-1β, and IL-6. The investigation has revealed that emodin provides protection to RTECs via suppressing NLRP3 inflammasome activation, thus reducing inflammatory processes and attenuating AKI caused by lipopolysaccharide. Lin et al. (2021) revealed chrysophanol has a protective effect against AKI, and it might alleviat H/R-induced lipid ROS accumulation and ferroptosis via regulating apoptosis, ER stress, and ferroptosis. Wang Y. et al. (2022) reported that emodin markedly improved I/R-induced renal dysfunction and reduced apoptosis in RTECs via the modulation of mitochondrial dynamics.

#### 4.1.5 Quercetin

Within kidney disease research, studies indicate that quercetin exhibits protective capabilities against radiation-induced DNA damage and kidney cell death (Özyurt et al., 2014; Zhang N. et al., 2024), while simultaneously mitigating AKI via inflammatory suppression and enhancement of antioxidant systems and SIRT1 (Khajevand-Khazaei et al., 2018). Additionally, quercetin has demonstrated protective effects against Cis-induced AKI *in vivo* (Sánchez-González et al., 2017) and has been shown to safeguard human renal proximal tubular cells against the toxicity of radiocontrast media *in vitro* (Andreucci et al., 2018). Significantly, recent investigations (Lu et al., 2018) illustrated that quercetin diminishes kidney injury by modulating the polarization of M1/M2 macrophages, thereby providing evidence that quercetin’s improvement of kidney inflammation and injury may be linked to macrophage function regulation. Tan’s research (Tan et al., 2020) established that quercetin reduces inflammatory mediator expression and release from macrophages, consequently alleviating renal damage in AKI. This mechanism is primarily associated with inhibiting Mincle and its associated signaling components Syk and NF-κB, which regulate macrophage polarization—specifically reducing pro-inflammatory M1 phenotypes while enhancing anti-inflammatory M2 populations. A study (Luo M. et al., 2022) indicated that quercetin reduces cellular damage and death, thereby limiting inflammatory processes in CI-AKI models through HIF-1α/lncRNA NEAT2/HMGB1 pathway inhibition. Gu’s research (Gu et al., 2021) emphasizes quercetin’s protective functions in COVID-19-related AKI through network pharmacological analyses and molecular docking investigations, identifying potential pathological mechanisms in coronavirus-induced renal damage. While quercetin’s complete regulatory mechanisms in COVID-19-associated AKI require further investigation, these findings provide valuable insights supporting quercetin’s potential development as a therapeutic intervention during the pandemic.

#### 4.1.6 Dihydroartemisinin

Recent research (Lei et al., 2021) indicated artesunate can mitigate renal damage and necroptosis, enhancing renal function and reducing inflammation. The underlying mechanism primarily inhibits macrophagic Mincle-mediated necroptosis and inflammation affecting tubular epithelial cells. In summary, artesunate acts by reducing M1 macrophage activation and suppressing the RIPK1/RIPK3/MLKL signaling cascade through the downregulation of Mincle expression, thereby diminishing the inflammatory response and necroptosis, which contributes to improvement of renal injury in AKI. Additionally, Liu’s investigation (Liu X. et al., 2019) illustrated that DHA may mitigate LPS-triggered AKI by suppressing NF-κB-mediated inflammation and inhibiting OS. Cheng et al. (2018) further demonstrated that DHA effectively improves S-AKI. Moreover, by preserving occludin expression, DHA prevents TNF-α induced hyperpermeability of the glomerular endothelium.

#### 4.1.7 Oridonin

Oridonin, an essential diterpenoid compound commonly found in traditional East Asian medicine, has gained significant recognition among medical researchers for its multiple therapeutic properties, encompassing anti-tumor, anti-inflammatory, antimicrobial, hepatic fibrosis prevention, and neurological effects (Xu et al., 2018; Xu et al., 2019). In a spontaneous lupus erythematosus mouse model, oridonin has been shown to diminish proteinuria and renal damage, while *in vitro* investigations have indicated that this compound reduces inflammatory cytokine production and the inflammatory cascade triggered by LPS following oridonin treatment (Tan et al., 2021). Furthermore, recent studies merging selenium nanoparticles and oridonin for esophageal cancer cell targeting reveal that oridonin enhances cell death by suppressing PI3K/AKT and Ras/Raf/MEK/ERK signaling cascades (Pi et al., 2017). Tan’s research (Tan et al., 2021) illustrated that oridonin exerts a notable anti-inflammatory effect and protects the kidneys during AKI, presumably through suppressing Mincle and its subsequent NF-κB and AKT signaling mechanisms. Additionally, Yan et al. (2020) reported that oridonin could mitigate I/R-AKI, presumably by suppressing macrophage inflammatory responses by inhibiting AKT-related signaling pathways.

#### 4.1.8 Neferine

Neferine (Nef), a bisbenzylisoquinoline alkaloid procured from the seed embryo of *N. Nucifera*, demonstrates various notable therapeutic characteristics, encompassing anti-tumor, antioxidant, and anti-inflammatory effects, as well as cardioprotective capabilities (Deng et al., 2017). It was revealed that Nef could alleviate Cis-induced AKI via autophagy stimulation (Li H. et al., 2017), suggesting that further confirmation and elucidation in animal models are warranted. Li H. et al. (2023) testified to Nef’s notable protective effects on Cis-induced AKI mice, partly attributed to autophagy activation. However, additional inquiries remain regarding the mechanisms by which Nef contributes to renal protection, particularly concerning autophagy’s function and its interactions with apoptosis, OS, and inflammation; further exploration of these mechanisms is essential for future research. Prior studies have established Nef’s renoprotective properties within kidney and vascular endothelium, potentially through the enhancement of autophagy (Li H. et al., 2017), inhibition of pyroptosis (Tang et al., 2019), or suppression of the inflammatory NF-κB pathway (Li H. et al., 2019). Xiong et al. (2024) demonstrated that Nef is capable of alleviating inflammation in by counteracting PPAR-α deficiency, thereby suppressing NF-κB pathway activation and inflammatory mediator production.

#### 4.1.9 Curcumin

Curcumin, the primary active compound of the plant Curcuma longa, has recently garnered attention for its potential renoprotective effects against AKI, encompassing glycerol-induced, gentamicin-induced, I/I-induced, and Cis-induced AKI (Fan et al., 2017; Wu et al., 2017). Notably, a recent study demonstrated that Curcumin mitigates inflammation triggered by titanium particles through regulating macrophage polarization (Li B. et al., 2017; Zhu et al., 2023). However, the exact mechanisms by which Curcumin influences AKI remain unclear, particularly whether it modulates macrophage polarization in AKI via the regulation of Mincle. One investigation (Ortega-Domínguez et al., 2017) assessed mitochondrial-related mechanisms associated with Curcumin’s protective effects in Cis-induced AKI by examining several parameters, including bioenergetics, ultrastructure, hydrogen peroxide (H_2_O_2_) generation, dynamics, SIRT3 protein levels, and mitophagy. Tan’s research (Tan et al., 2019) revealed that treatment with Curcumin significantly downregulated Mincle expression in infiltrated macrophages, alleviating renal inflammation mediated by M1 macrophages through a Syk/NF-κB-dependent mechanism. Zhu et al. (2020) established Curcumin’s protective function in both cellular and mouse models of S-AKI, partially attributing its mechanism to decreased inflammatory mediator production through suppression of JAK2/STAT3 and TNF-α signaling pathways.

#### 4.1.10 Celastrol

Celastrol, a bioactive triterpenoid derived from Tripterygium wilfordii Hook. F exhibits notable biological activities, encompassing anticancer and anti-inflammatory effects (Lu et al., 2021). Celastrol has reportedly alleviated CKD by upregulating cannabinoid receptor 2 (Tang et al., 2018). Furthermore, the targeted delivery of Celastrol to glomerular endothelium and podocytes can potentially enhance renal function (Wu Q. et al., 2022). The function of Celastrol in AKI is increasingly being elucidated, with findings indicating that it can improve Cis-induced AKI by suppressing NF-κB and enhancing mitochondrial function (Yu et al., 2018). Celastrol has also been effective against mesangioproliferative glomerulonephritis (Guo et al., 2017), recent studies (Chu et al., 2014) have additionally reported that Celastrol ameliorates IR-AKI, which correlates with inhibiting NF-κB activation and inflammation. However, the impact of Celastrol on Cis-induced AKI remains to be thoroughly investigated. Another investigation (Yu et al., 2018) demonstrated that Celastrol could mitigate Cis-induced AKI by antagonizing NF-κB-mediated inflammation and safeguarding mitochondrial function. These findings strongly suggest that Celastrol possesses translational potential as a natural therapeutic agent for treating Cis-induced AKI in clinical settings. Pan et al. (2023) found cisplatin-induced ferroptosis leads to tubular cell injury and renal dysfunction, characterized by lipid peroxidation. The upregulation of Nrf2 by Celastrol markedly enhances GPX4 expression, thereby preserving renal function and redox homeostasis.

#### 4.1.11 Embelin

Embelin, a benzoquinone derivative extracted from Embelia ribes, exhibits potential antibacterial, antidiabetic, antioxidant, analgesic, antifertility, and anticancer properties (Ning et al., 2018). Research indicates that Embelin functions as an inhibitor of NF-κB signaling, thereby suppressing NF-κB-regulated anti-apoptotic and metastatic gene products. Additionally, it may serve a regulatory function in immune cells during acute liver injury and allergic asthma (Wang H. et al., 2019; Azman et al., 2021). Nonetheless, the precise mechanisms and significance of Embelin in S-AKI remain unclear. Tang Q. et al. (2023) investigated the immunomodulatory and anti-inflammatory properties of Embelin in LPS- induced AKI, revealing that Embelin can mitigate AKI by inhibiting M1 macrophage activation and blocking NF-κB signaling in mice.

#### 4.1.12 Gastrodin

Gastrodin (GAS), the primary active component derived from G. elata Blume, has been clinically employed to treat patients experiencing vertigo (Lai et al., 2022; Chen X.-Y. et al., 2023). Observations suggest that GAS can restore GPX4 expression in two models of OS (Jiang et al., 2020). Current findings (Qiu et al., 2024) demonstrate that FOXO3A regulates the transcription of GPX4. Additionally, GAS has been shown to activate SIRT1, thereby protecting Cis-induced AKI by suppressing ferroptosis through the SIRT1/FOXO3A/GPX4 signaling pathway.

#### 4.1.13 Isorhamnetin

Isorhamnetin is a flavonoid compound derived from various plants, encompassing ginkgo biloba and sea-buckthorn, and demonstrates diverse therapeutic activities. These effects include immunomodulatory, antiviral, antioxidant, anti-inflammatory, anti-tumor, and serum cholesterol reduction properties (Tian et al., 2021). Recent studies demonstrated isorhamnetin possesses significant anti-inflammatory effects on macrophages, including regulating macrophage inflammatory responses in conditions such as cord injury, atherosclerosis, and osteoarthritis, leading to M1 macrophage inhibition and M2 macrophage upregulation (Chen et al., 2021). However, the effect of isorhamnetin on AKI remains unknown, and whether isorhamnetin can suppress M1 macrophage activation and promote M2 macrophage in AKI kidneys through the regulation of SLPI needs further investigation. A study (Jian et al., 2024) clarified that isorhamnetin markedly upregulated SLPI to inhibit the Mincle/Syk/NF-κB signaling pathway, which diminished M1 macrophage differentiation while facilitating M2 macrophage differentiation to attenuate AKI-triggered renal inflammatory reactions.

#### 4.1.14 Magnesium lithospermate B

Salvia miltiorrhiza Bunge exhibits a range of pharmacological activities, including promoting blood circulation, mitigating OS damage, and preventing apoptosis (Gao F. et al., 2019). Magnesium lithospermate B (Mlb), the primary water-soluble active component of Salvia miltiorrhiza Bunge, contributes to managing cardiovascular diseases and offers protective effects against renal disorders. In renal ablation/infarction models, significant reductions in renal injury and apoptosis have been attributed to Mlb (Wang M. et al., 2019). Salvianolate, a compound derived from Salvia miltiorrhiza extracts and primarily composed of salvia magnesium acetate, has been demonstrated to mitigate contrast-induced AKI while alleviating OS associated with podocyte injury (Liang et al., 2021). Our findings (Shen et al., 2022) indicate that Mlb treatment lessens the severity of CI-AKI by suppressing ROS generation, apoptosis, and mitochondrial damage by modulating Drp1 levels.

#### 4.1.15 Shikonin

Peng’s study (Peng et al., 2022) showed that shikonin could improve cecal ligation and perforation-induced AKI and LPS-induced dysfunction of RTECs. Besides, the mechanism of apoptosis, OS, and inflammatory response may be partially linked to modulating the NOX4/PTEN signaling pathway.

#### 4.1.16 Liquiritigenin

Studies have demonstrated that liquiritigenin possesses diverse biochemical and pharmacological attributes, which include hepatoprotective, anti-hyperlipidemic, antioxidant, anti-inflammatory, and anticancer effects (Jain et al., 2022). Evidence indicates licorice could alleviate cisplatin-induced hepatotoxicity and nephrotoxicity through mechanisms involving anti-apoptosis, OS reduction, anti-inflammatory actions, and enhanced metabolic processes (Man et al., 2020). Furthermore, liquiritigenin was observed to amplify the suppressive impact of cisplatin on invasion and metastasis by downregulating MMP-2/9 and modulating the PI3K/AKT signaling pathway (Shi et al., 2015). However, the effects and underlying mechanisms of liquiritigenin concerning Cis-induced AKI remain to be elucidated. Zhou’s study (Zhou M. et al., 2022) confirmed that liquiritigenin could function as a nephroprotective agent against Cis-induced AKI by enhancing mitochondrial function.

#### 4.1.17 Oroxylin A

A study (Yao M. et al., 2022) has demonstrated the therapeutic potential of Oroxylin A (OA), a principal active constituent of *Scutellaria baicalensis*, in addressing AKI and its progression to CKD. Mechanistically, it was shown that OA markedly ameliorated mitochondrial injury induced by hypoxia-reoxygenation through the enhancement of the PPARα-BNIP3 signaling pathway. Consequently, therapeutic strategies that utilize OA or target the PPARα-BNIP3 axis to regulate mitochondrial homeostasis may present innovative approaches for managing the transition from AKI to CKD.

#### 4.1.18 Arbutin

Previous studies have reported that arbutin (Ar), isolated from Chinese yam, exhibits a concentration of 0.08‰ in extracts measured through liquid chromatography-mass spectrometry (LC-MS) (Zeng et al., 2018). Data derived from NP analysis indicated that Ar potentially mitigates AKI by demonstrating anti-inflammatory properties and modulating the Akt signaling pathway. Recent findings (Zhang et al., 2021b) indicated that Ar could confer protection against LPS-induced AKI by inhibiting inflammation and apoptosis through the Akt signaling pathway, thereby establishing a molecular foundation for innovative therapeutic strategies for AKI.

#### 4.1.19 Puerarin

Puerarin, derived from Radix puerariae (R. puerariae), has garnered recent interest due to its diverse pharmacological properties for treating kidney disorders, including AKI and CKD (Ma et al., 2014; She et al., 2014). Additionally, Ma et al. reported that puerarin might alleviate CI-AKI (Ma et al., 2017). Nonetheless, the renal protective mechanisms of puerarin in CI-AKI remain poorly understood. Wu et al. (2020) demonstrated that puerarin could alleviate CI-AKI by downregulating miR-31 expression, enhancing Numb activation, and subsequently inhibiting the Notch signaling pathway.

#### 4.1.20 Isoorientin

Previous research (Anilkumar et al., 2017) has demonstrated that Isoorientin (Iso) exhibits multiple pharmacological effects, including anti-inflammatory and antioxidant activities. Furthermore, it has been established that Iso mitigates APAP-induced hepatotoxicity by stimulating the Nrf2 antioxidative pathway and engaging AMPK/Akt/GSK3β (Fan et al., 2018). Natural products serve as primary activators of Nrf2, capable of modulating the Nrf2/ARE pathway to alleviate OS, thus garnering increasing scholarly interest in recent years. Fan’s investigation (Fan et al., 2020) offers an extensive overview of Iso’s therapeutic promise in Cis-induced AKI and its mechanistic pathways. The findings suggest that the lack of Nrf2 exacerbates Cis-induced AKI, and the pharmacological activation of Nrf2 may provide a novel therapeutic approach to avert kidney damage.

### 4.2 Saponins

#### 4.2.1 Astragaloside IV

Astragaloside IV (AS-IV), a key active constituent procured from Astragalus membranaceus, is associated with a multitude of pharmacological functions (Zhang D. Q. et al., 2018; You et al., 2019) and has been evidenced to offer protective effects in diverse models of kidney disease (Feng et al., 2022). Gui et al. (2013) established that AS-IV mitigated structural and biochemical abnormalities while also inhibiting OS and apoptosis in rats suffering from AKI. Tang et al. (2022) demonstrated that AS-IV protect S-AKI in RTECs by strengthening the PI3K/AKT pathway. Recent research (Guo et al., 2023) has indicated that AS-IV mitigates ferroptosis during I/R-AKI, confirmed through NP, molecular docking, MD simulation, and experimental validation. It has been demonstrated that AS-IV reduces ROS and Fe^2+^ levels while promoting the expressions of GPX4 and SLC7A11 in OGD/R-injured HUVECs, thereby effectively restraining ferroptosis in I/R-AKI. Other investigations (Shi et al., 2021; Zhang M. et al., 2022) propose an alternative mechanism for AS-IV in I/R-AKI cases. Prior reports have established that AS-IV can modulate MAPK, NF-κB, and Nrf2 expression to mitigate renal injury. However, the specific impact of AS-IV on HHcy-exacerbated S-AKI remains inadequately understood. A recent study (Xu et al., 2024) revealed that TPL2 elevation in HHcy-intensified S-AKI occurs through Gpr97 pathway activation, mechanistically amplifying inflammatory responses and cellular mortality. Moreover, initial findings demonstrated AS-IV’s ability to minimize Hcy-intensified S-AKI through suppression of Gpr97-TPL2 signaling cascades. Ast potentially guards against AKI by strengthening multiple metabolic processes, encompassing amino acid pathways, glyoxylic acid, dibasic acid metabolism, glutathione processes, and UFA synthesis. These mechanisms potentially connect to inflammatory control, metabolic enhancement, and OS suppression (Song et al., 2021). Additionally, Ast has the capability to inhibit expression of NLRP3 inflammasomes and restrict proinflammatory mediator release through autophagy induction (Qu et al., 2019).

#### 4.2.2 Frehmaglutin D and rehmaionoside C

Violetone substances Frehmaglutin D and rehmaionoside C, extracted from Rehmannia glutinosa, demonstrate estrogenic properties in combating S-AKI. Research indicates that these compounds potentially achieve their beneficial impact on S-AKI via the ER-TLR4-IL-1β signaling cascade, leading to enhanced regulation of inflammation, apoptosis, and OS (Liu M. et al., 2024). Both substances exhibit activity through ERα and ERβ receptors, showing comparable mechanistic pathways. Furthermore, studies have established that ERα and ERβ can form direct or indirect associations with TLR4, and these interactions vary based on concentration levels.

#### 4.2.3 Ginsenoside Rg1

Ginsenoside Rg1 (Rg1) is an active compound procured from Panax ginseng, exhibiting diverse therapeutic effects, encompassing immunoregulatory, anti-inflammatory, antioxidant, anti-apoptotic, neuroprotective, and cardioprotective activities (Chen J. et al., 2019). Notably, numerous studies (Mao et al., 2016; Guo et al., 2019) revealed Rg1 alleviates podocyte damage caused by angiotensin II, experimental glomerular nephritis, and glomerular fibrosis. Evidence provided by Hu et al. (2024) supports the advantageous effects of Rg1 in S-AKI model. Specifically, Rg1 reduces renal damage and decreases cell apoptosis, oxidative damage, and inflammation in S-AKI mice by modulating SIRT1/NF-κB signaling pathway.

#### 4.2.4 Polydatin

Polydatin (PD), a natural bioactive compound extracted from Polygonum cuspidatum Sieb. et Zucc.‘s dried roots, exhibit potential therapeutic benefits in diverse renal conditions. Studies indicate that PD operates through multiple mechanisms, encompassing oxidative stress reduction, inflammation suppression, fibrosis prevention, mitochondrial function enhancement, and autophagy modulation (Sun and Wang, 2020; Peritore et al., 2021). *In vitro* studies demonstrate PD effectively blocks the production of inflammatory cytokines (Lou et al., 2015). Additionally, previous investigations have established that PD provides protective effects against AKI induced by cecal ligation and puncture, as well as renal I/R injury in mice (Gao et al., 2015; Meng et al., 2016). The compound substantially shields mice from AKI by modulating Scr, BUN, and inflammatory mediator levels. This protection stems from its dual action of suppressing NF-κB pathway activity while enhancing Nrf2 signaling (Gu et al., 2019). Contemporary research (Huang et al., 2021) suggests PD’s ability to suppress ferroptosis, leading to improvements in myocardial I/R damage and cerebral trauma. Yet, PD’s specific influence on Cis-induced AKI remains to be fully elucidated. Findings from one investigation (Zhou L. et al., 2022) highlight PD’s significant renoprotective impact against ferroptosis in Cis-induced AKI models, achieved through multiple mechanisms: limiting excessive cellular iron accumulation, decreasing ROS generation, preserving GSH levels, boosting GPX4 functionality, thus minimizing lipid oxidation and ferroptotic susceptibility, ultimately decelerating AKI progression.

#### 4.2.5 Tiliroside

Tiliroside (Tili) is a natural flavonoid frequently found in various plants and exhibits numerous biological activities, encompassing anti-inflammatory and antioxidant properties (Grochowski et al., 2018). The renoprotective effects of Tili on LPS-induced AKI have been emphasized by Yi et al. (2023), who noted its ability to suppress inflammation, OS, and tubular cell apoptosis while promoting autophagy flux through a shift towards the intrarenal ACE2/Ang1-7 axis and away from the intrarenal ACE/Ang II axis. Additionally, Cai’s study (Cai et al., 2024) demonstrated that Tili triggered Nrf2 activation through disruption of Keap1-Nrf2 interaction, indicating that tiliroside administration significantly safeguarded mice from Cis- and I/R-induced AKI. The protective outcomes of Tili on renal tissue were achieved through ferroptosis suppression via Nrf2 pathway activation.

#### 4.2.6 Paeoniflorin

Paeoniflorin (PF), isolated from Paeonia lactiflora Pal, is associated with diverse biological effects (Xing et al., 2023), including antioxidative, anti-inflammatory, anti-apoptotic, analgesic, and immunomodulatory properties. Recently, it was figured out by Wen et al. (2019) that PF mitigates the impairment of autophagy flux caused by intestinal I/R via LKB1/AMPK pathway activation. Research has indicated PF’s capacity to reduce acute necrotizing pancreatitis-linked AKI by suppressing inflammatory responses and kidney cell death (Wang et al., 2016). A study (Xing et al., 2023) demonstrated PF prevents HK-2 cells from hypoxia-reoxygenation (H/R) injuries, suggesting its protective mechanism involves Nrf2-dependent antioxidant pathways. The Nrf2/HO-1 signaling cascade emerges as a crucial therapeutic target in H/R-induced OS management. PF’s cell-protective and antioxidant capabilities show promise for clinical applications in I/R-induced AKI treatment. Ma et al. (2023) indicated that PF reduced serum biochemical markers, histological damage, ferroptosis, and inflammation in I/R- AKI mice. Additionally, ferroptosis and inflammation triggered by H/R were inhibited by PF in HK-2 cells. RNA sequencing analysis suggested PF inhibits ferroptosis in HK-2 cells by enhancing SLC7A11 following H/R exposure. Consequently, these findings suggest PF prevention of ferroptosis by AKI depends on SLC7A11. Zhang M. Y. et al. (2023) utilized an integrated network pharmacological approach alongside RNA-Seq methods to investigate transcriptional alterations triggered by PF in Cis-induced AKI, revealing that PF reduces cellular death and inflammatory responses in Cis-induced AKI via enhancement of Hsp90AA1-Akt protein-protein interactions.

#### 4.2.7 Hyperoside

Hyperoside (Hyp), a flavonol glycoside compound, has been indicated to enhance the progression of kidney diseases. Wu et al. (2019) demonstrated that Hyp protects against I/R-induced tubular cell injury through its influence on mitochondrial fission, OS, and apoptosis. Notably, Hyp targets the OMA1-OPA1 system to prevent mitochondrial fragmentation, thereby promoting tubular cell survival. Pre-treatment with Hyp has markedly reduced apoptosis and OS in cases of I/R AKI (Wu et al., 2019). Furthermore, Hyp mitigates Cis-induced AKI by inhibiting the NLRP3 inflammasome, mediated through ROS/MAPK/NF-κB signaling pathway (Li Z. et al., 2023). Additionally, Hyp inhibits AMPK-ULK1-mediated autophagic activity, which attenuates D-galactose-induced renal aging and injury (Liu B. et al., 2018). Moreover, Hyp pre-treatment markedly reduces proteinuria in diabetic mice and protects the glomerular basement membrane from OS and damage (An et al., 2017). The impact of Hyp on Cis-induced AKI in rats requires further investigation. Yuan et al. (2023) found that Hyp could enhance OAT1 expression through HNF-1α and PXR regulation, thereby improving OAT1 uptake capacity, lowering indoxyl sulfate accumulation *in vivo*, and facilitating its urinary elimination, consequently reducing Cis-induced AKI.

#### 4.2.8 Monotropein

Monotropein is characterized by diverse pharmacological activities, encompassing antioxidant, anti-inflammatory, and anti-apoptotic effects (Zhu et al., 2016). Zhang et al. (2020a) demonstrated that monotropein mitigates cisplatin-induced nephrotoxicity while decreasing Scr and BUN levels. Additionally, monotropein effectively inhibits cisplatin-induced OS by lowering MDA levels and boosting GSH, SOD, and CAT enzymatic functions. The protective effects of monotropein against cisplatin-mediated AKI operate through multiple pathways: it stimulates the Nrf2/HO-1 cascade to counter OS, blocks NF-κB signal transduction to reduce inflammatory responses, and modulates the expression of apoptosis-related proteins in this kidney damage model.

#### 4.2.9 Forsythiaside A

Modern pharmacology demonstrates that Forsythiaside A (FTA), derived from Forsythia Fructus, exhibits various pharmacological effects, including antibacterial, antioxidant, antiviral, hepatoprotective, anti-inflammatory, and neuroprotective properties (Chen Y. et al., 2023). However, limited studies have been conducted regarding the protective effects of FTA on the kidneys and its potential to ameliorate renal damage in the context of S-AKI. Chen Y. et al. (2023) developed an LPS-induced AKI model and discovered FTA provided protection to renal tissue during sepsis initiation, thereby diminishing kidney inflammation and cell death. Additionally, FTA has emerged as a promising suppressor of ER stress-associated apoptosis, with its regulatory mechanisms operating partly through PERK pathway suppression.

#### 4.2.10 Salidroside

Salidroside (SA), a bioactive compound of Rhodiola rosea, is celebrated for its extensive biological properties encompass anti-inflammatory, antioxidative, anti-tumorigenic, and anti-radiation effects (Rong et al., 2020). A multitude of investigations have established the protective benefits of SA against various organ damages, mainly through the mitigation of OS, the inhibition of apoptosis, the reduction of intracellular calcium overload, and the enhancement of mitochondrial function (Zhang P. et al., 2023). Tang Z. et al. (2023) demonstrated SA augmented the activity of SODs via activating PI3K/AKT signaling pathway, resulting in the elimination of ROS, attenuation of OS injuries and ferroptosis, thereby safeguarding renal function.

#### 4.2.11 Notoginsenoside Fc

A recent report has indicated Notoginsenoside Fc (Fc) facilitates re-endothelialization acceleration after vascular injury in diabetic rats by promoting autophagy (Liu J. et al., 2019). It has been observed Fc mitigates injury to vascular endothelial cells through modulating PPAR-γ-mediated pathway in diabetic rats (Liu J. et al., 2018). Furthermore, another study has demonstrated (Wei et al., 2022) Fc reduces tubular injury and alleviates mitochondrial dysfunction in AKI mice, in part through the modulation of the SIRT3/SOD2 pathway.

#### 4.2.12 Capilliposide A

Fang’s study (Fang et al., 2024) indicated that the extract of Lysimachia capillipes Hemsl, known as Capilliposide A, may be an effective therapeutic agent against Cis-induced AKI. This effect is thought to arise from a synergistic interaction with endogenous metabolites linked to amino acid metabolism and ER stress, achieved by suppressing PERK-ATF4-CHOP-mediated apoptosis and OS.

#### 4.2.13 Gypenoside XVII

Gypenoside XVII (GP-17), a tetracyclic triterpenoid saponin extracted from Gynostemma pentaphyllum, exhibits multiple pharmacological benefits against disorders affecting cerebrovascular, cardiovascular, and skin systems. Previous studies have revealed that GP-17 offers protection from myocardial I/R injury through the suppression of ER stress-related protein expression, including GRP78 and CHOP, thus reducing ER stress (Yu et al., 2021; Su et al., 2022). Research has also established that GP-17 reduces mitochondria-linked apoptotic processes in myocardial tissue (Lee et al., 2019). Studies by Wang J. et al. (2024) established GP-17 attenuates ER stress during renal I/R while suppressing NLRP3 inflammasome activation, consequently inhibiting pyroptosis and delivering anti-inflammatory benefits.

#### 4.2.14 Icariin

Icariin could alleviate Cisp-induced AKI, primarily through downregulation of TNF-α levels and inhibition of NF-κB and apoptosis-related proteins (Ma et al., 2015). Additionally, icariin has exhibited effectiveness in decreasing S-AKI-related mortality by lessening oxidative injury, regulating inflammatory responses, and disrupting proapoptotic pathways (Xie et al., 2018). Zhang D. et al. (2024) examined the pathological mechanisms of FA-induced AKI and icariin’s protective role using proteomic analysis. Their research revealed complement and coagulation cascade pathways play significant roles in AKI development and progression, and icariin attenuates AKI by suppressing these signaling cascades.

#### 4.2.15 Hederagenin

Recent studies have indicated Hederagenin (HDG) can both attenuate cerebral I/R injury through the regulation of MLK3 signaling pathway (Yu et al., 2020) and enhance renal fibrosis by targeting muscarinic acetylcholine receptors (Yang and He, 2022). Xie’s research (Xie et al., 2022) identified lncRNA-A330074k22Rik in kidney tissues of Cis-induced AKI and highlighted its crucial role in the pathogenesis and progression of AKI, where it exerts a promoting effect. Furthermore, HDG protects against Cis-induced AKI and LPS-induced inflammatory injury in RTECs. Mechanistically, the inhibition of lncRNA A330074k22Rik by HDG markedly suppresses the Axin2/β-catenin pathway, thereby downregulating the inflammatory response associated with AKI.

#### 4.2.16 Trilobatin

Duan et al. (2022) developed a Cis-induced AKI model in mice. Subsequently, they assessed the protective role of Trilobatin (TLB) pretreatment against renal toxicity by inhibiting oxidative damage and apoptosis. Observations indicated that the parameters in the TLB treatment group exhibited varying degrees of improvement compared to the control group, with the 100 mg/kg dosage showing superior protective effects, thus signifying a dose-dependent response to TLB administration. The current investigation demonstrated that TLB pretreatment markedly reduced apoptosis induced by cisplatin.

#### 4.2.17 Isoacteoside

Previous research has indicated that the inflammatory response associated with severe acute pancreatitis (SAP) might be diminished by inhibiting TLR4/NF-κB signaling pathway (Wang et al., 2017). Wang B. et al. (2021) established rat models of SAP to examine anti-inflammatory properties of isoacteoside in SAP-induced AKI. Isoacteoside was found to mitigate AKI resulting from SAP by reducing inflammation. Consequently, isoacteoside could serve as a potential therapeutic agent for both SAP and SAP-induced AKI. This mechanism may involve inhibiting TLR4/NF-κB p65 signaling pathway.

#### 4.2.18 Oleuropein

Cui et al. (2021) indicated Oleuropein (OP) exerted anti-inflammatory effects via the NF-κB/MAPK signaling pathway by suppressing the dimerization of TLR4. These effects of OP may contribute to its capability to ameliorate LPS-associated AKI by modulating the TLR4-MyD88-NF-κB/MAPK axis.

#### 4.2.19 Loganin

Loganin is an iridoid glycoside derived from Corni fructus, known for its use in replenishing liver and kidney functions while suppressing sweating and seminal emissions. A study (Kim et al., 2021) demonstrated that loganin displayed reno-protective properties against Cis-induced AKI by inactivating ERK 1/2. These findings suggest that loganin may be an effective adjuvant in cisplatin-based cancer therapies.

#### 4.2.20 Nodakenin

Nodakenin, a furanocoumarin glycoside isolated from Peucedanum decursivum Maxim, has recently been shown to enhance progressive fibrosis by mediating the expression of Snail1 (Li et al., 2020). Liao’s research (Liao et al., 2021) highlighted that nodakenin markedly inhibited I/R-induced AKI in mice and hypoxia-treated primary RTECs by modulating the activation of the NF-κB and ROS-induced NLRP3 inflammasome, thereby improving inflammation in I/R-AKI.

#### 4.2.21 Arginyl-fructosyl-glucose

Arginyl-fructosyl-glucose is one of the key non-saponins present in red ginseng, which, along with saponins, is recognized for its strong protective effects against kidney injury (Kim et al., 2014). Li R. Y. et al. (2019) found AFG mitigated the side effects of Cis-induced AKI mice, partly by restoring antioxidative activity and reducing the inflammatory response. Notably, AFG pretreatment enhanced recovery from renal injury by alleviating OS, mediating NF-κB-related inflammation, and inhibiting the PI3K/Akt apoptotic signaling pathways.

#### 4.2.22 Eleutheroside B

Eleutheroside B has been reported to exhibit a range of pharmacological activities, including anti-inflammatory and anti-radiation effects. Zang’s study (Zang et al., 2019) found that eleutheroside B offers protection against Cis-induced AKI in mice, mitigating the damage caused by cisplatin exposure and hypoxia-reoxygenation in HK-2 cells. It has been shown that eleutheroside B inhibits the expression of KIM-1, reduces inflammation, and prevents both apoptosis and programmed necrosis. The underlying mechanism may involve the activation of the IGF pathway and its downstream signaling by downregulating IGFBP-7 expression, thereby promoting cellular proliferation.

#### 4.2.23 Esculentoside A

Esculentoside A (EsA), derived from the root of Phytolaca esculenta, has been noted for its anti-inflammatory and antioxidant properties. Chen’s study (Chen et al., 2017) demonstrated the protective effects of EsA against LPS-induced AKI in mice. It was observed that EsA protects against LPS-induced AKI by inhibiting the inflammatory response through the activation of PPAR-γ.

### 4.3 Flavonoids

#### 4.3.1 Tanshinone IIA

Tanshinone IIA represents a bioactive compound extracted from Salvia miltiorrhiza, which has been extensively utilized in treating various ailments throughout Asia. Jiang et al. (2016b) revealed that Tanshinone IIA mitigated the overactivity of glycogen synthase kinase (GSK)3β, as well as the hyperactivation of its downstream mitogen-activated protein kinases, which are fundamentally involved in renal fibrogenesis and inflammatory processes. The inhibition of GSK3β is likely a pivotal mechanism through which the therapeutic efficacy of Tanshinone IIA is mediated, as sodium nitroprusside, a known GSK3β activator, markedly counteracts its renoprotective benefits. Additionally, another investigation (Jiang et al., 2016a) demonstrates that Tanshinone IIA reduces kidney damage following folic acid exposure in a murine model. This compound mitigates damage to RTECs, facilitates recovery, alleviates the inflammatory response, and obstructs the progression of long-term kidney fibrosis. Mitochondrial dysfunction can be interpreted as a direct pathophysiological link between kidney-lung interactions during the phases of AKI and ALI triggered by renal IR. Renal IR is known to provoke mitochondrial dysfunction and apoptosis within myocardial cells. Tanshinone IIA, in combination with cyclosporine A, is viewed as a protective agent that diminishes lung apoptosis through the modulation of mitochondrial function by activating PI3K/Akt/Bad pathway (Tai et al., 2021b; Tai et al., 2022). In the research of Tai et al. (2021a), mitochondria were isolated from rat myocardial tissues, establishing that mitochondrial dysfunction within the myocardium occurred alongside renal IR, subsequently leading to myocardial cell apoptosis, which was worsened by obesity. Dou et al. (2022) employed network pharmacological analysis to investigate target genes and regulatory networks associated with the effects of Salvia miltiorrhiza in AKI treatment. Complementary experiments using an *in vivo* AKI mouse model and *in vitro* methodologies were conducted to explore the renal protective properties of Tanshinone IIA. Tanshinone IIA may enhance renal inflammation attenuation by inhibiting PXR-mediated NF-κB activation.

#### 4.3.2 Shionone

Shionone is a natural constituent derived from the dried rhizome of Aster tataricus L. f., exhibiting anti-inflammatory properties (Wang X. et al., 2021). Prior investigations have indicated the administration of LPS to animal models or cellular systems may elicite an inflammatory response analogous to that observed in clinical sepsis (Zhang et al., 2020b). Zhang et al. (2021a) proposed that sepsis induced AKI leads to the production of M1 macrophages, which may function in the inflammatory response associated with AKI. By inhibiting ECM1 and activating the GM-CSF/STAT5/Arg1 pathway to promote the differentiation of alternative macrophage M2, Shionone effectively diminishes the inflammatory response, thereby facilitating tissue repair and mitigating AKI.

#### 4.3.3 5-O-methyldihydroquercetin and cilicicone B

5-O-methyldihydroquercetin (GS1) and cilicicone B (GS2) were identified as the two predominant flavonoids extracted from the plant; however, their pharmacological activities remain underexplored. Zeng M. et al. (2020) discovered that GS1 and GS2 exhibit significant anti-inflammatory and antioxidant activities, markedly alleviating renal damage. It is suggested that GS1 and GS2 may exert their effects by inhibiting TLR4/MyD88/TRIF/NLRP3 signaling pathway.

#### 4.3.4 Zingerone

Zingerone, a phenolic alkanone extracted from ginger, has been noted for its diverse pharmacological properties. Prior investigations have indicated that zingerone demonstrates anti-inflammatory effects by inhibiting the NF-κB signaling pathway (Hsiang et al., 2015). It has been reported that zingerone treatment mitigates activation of NF-κB in models of acute lung injury induced by LPS (Xie et al., 2014). Research conducted by Song et al. (2016) revealed that a dose of 10 mg/kg of zingerone could attenuate LPS-induced expression of TLR4, although this inhibition was relatively modest. Notably, zingerone markedly suppressed LPS-induced NF-κB activation. This study further established that zingerone possesses protective effects against LPS-induced AKI. The promising anti-inflammatory mechanism of zingerone is attributed to its capacity to inhibit TLR4-mediated NF-κB activation and the inflammatory response.

### 4.4 Polyphenols

#### 4.4.1 Salvianolate

Salvianolate (SAL) is known for its ability to scavenge free radicals, exert anti-OS effects and inhibit thrombosis. Its efficacy in treating conditions such as coronary heart disease, angina pectoris, diabetes, and other ailments has been well-established (Yang et al., 2021). Furthermore, clinical studies have demonstrated that salvianolate exhibits both effectiveness and safety in patients with diabetic nephropathy (Qi et al., 2007). Sun D. et al. (2022) indicated that SAL facilitates Nrf2 activation and promotes the expression of its downstream target genes, which markedly contributes to reducting ROS levels within cells. Additionally, SAL impacts the thermal stability of Keap1, with modifications to the Cys151 residue of Keap1 being crucial for Nrf2-dependent transcriptional activation mediated by SAL.

As a multitarget agent, salvianolic acid A (SAA) demonstrates significant potential in treating kidney disorders (Yao L. et al., 2022). Zhang Z. et al. (2018) suggest that SAA may confer protection against I/R-AKI, possibly due to its capacity to mitigate damage to PTC endothelium and to preserve PTC integrity, thereby alleviating hypoxia in the vicinity of renal tubules and ameliorating acute tubular necrosis. Recent investigations have indicated that SAA exerts anti-inflammatory and anti-OS effects by activating the Akt/GSK-3β/Nrf2 and inhibiting the NF-κB signaling pathway in 5/6Nx rats (Zhang et al., 2019). Moreover, in LPS-induced AKI, SAA enhances renal function by inhibiting the activation of the TLR4/MyD88 signaling pathway, subsequently reducing the release of inflammatory mediators (Zeng X. et al., 2020). Diao et al. (2023) reported that SAA improved gentamicin-induced AKI and 5/6Nx-induced CKD, likely through the inhibition of inflammatory factor release, alleviation of OS injury, and modulation of the MAPK and TGF-β1/Smad signaling pathways. Prior investigations have indicated that salvianolic acid B (SalB) mitigates injuries in various organs and maintains redox homeostasis, particularly the balance of ROS (Tang et al., 2014). SalB confers protection by enhancing the Nrf2 antioxidant signaling pathway in animal models (Liao et al., 2020). Pang et al. (2020) revealed that the primary mechanism by which SalB improves AKI involves inhibiting NLRP3 activation through direct stimulation of nuclear Nrf2 expression, subsequently reducing pyroptosis. Prior investigation has shown that salvianolic acid C (SalC) diminishes inflammation, OS, and caspase-mediated apoptosis by inactivating the Keap1/Nrf2/HO-1 signaling pathway in AKI (Uzunoglu et al., 2011; Mercantepe et al., 2018). A study (Chien et al., 2021) demonstrated that SalC regulates inflammatory responses in Cis-induced AKI animal model by suppressing renal histopathological changes, inflammatory cell infiltration, and the release of proinflammatory cytokines. SalC presents potential as a therapeutic agent, providing robust anti-inflammatory and antioxidant effects against AKI, mediated by the inhibition of signaling axes involving TLR-4, NF-κB, MAPK, HO-1, and Nrf2.

#### 4.4.2 Honokiol

Honokiol, isolated from Magnolia officinalis, exhibits anti-inflammatory and antioxidant properties. Honokiol may inhibit OS and inflammation associated with renal I/R injury (Yu et al., 2016). The current investigation (Xia et al., 2019a) has demonstrated that honokiol decreases iNOS, NO, and MPO levels *in vitro* experiments. Conversely, the activities of GSH and SOD exhibit significant increases following honokiol treatment. Additionally, honokiol enhances the antioxidant capacity of HO-1 in rats subjected to CLP and ameliorates the morphological alterations in the kidneys of these rats. ZnPPIX, an inhibitor of HO-1, can diminish the antioxidant effect of honokiol. Collectively, these findings indicate that honokiol alleviates OS in sepsis-induced AKI. Moreover, protein analysis of TLR2, TLR4, TRIF, MyD88, IκBα, and p-IκBα reveals that honokiol can inhibit the aberrant activation of the TLR signaling pathway.

### 4.5 Lipoid

#### 4.5.1 Artesunate

Beyond its anti-malarial properties, many investigations report artesunate exhibits considerable anti-inflammatory, antioxidant, and anti-autophagy characteristics. A recent study has indicated that the effect of artesunate in mitigating ulcerative colitis is linked to its ability to alleviate excessive ER stress-mediated intestinal barrier impairment and the inflammatory response (Yin et al., 2021). Furthermore, Lei et al. (2021) revealed that artesunate markedly diminishes renal damage and necroptosis, enhancing renal function and inflammation. The underlying mechanism is primarily associated with inhibiting macrophage Mincle-mediated necroptosis and the inflammatory response directed towards tubular epithelial cells. In summary, artesunate inhibits the activation of M1 macrophages and the RIPK1/RIPK3/MLKL signaling cascade by down-regulating Mincle expression, consequently reducing both the inflammatory response and necroptosis, thereby ameliorating renal injury in AKI.

#### 4.5.2 Limonin

In various medical contexts, limonin exhibits numerous biological activities, such as antibacterial, anti-inflammatory, antioxidant, and antiproliferative effects (Duan et al., 2021; Luo J. et al., 2022). Zhou et al. (2023) have stated that limonin acts as an ERK2 agonist, possessing the ability to confer protection against ischemic AKI. The findings clearly illustrated that limonin reduces cellular mortality and facilitates tubule repair and regeneration by activating ERK.

#### 4.5.3 Parthenolide

A sesquiterpene lactone known as parthenolide (PTL) is derived from the perennial plant feverfew and has demonstrated anti-inflammatory properties (Freund et al., 2020). According to Shou et al. (2023), PTL has the capability to modulate inflammatory factors in cases of AKI and mitigate CLP-induced sepsis through the NF-κB p65 signaling pathway.

#### 4.5.4 Alisol B 23-acetate

Luan et al. (2021) reported that Alisol B 23-acetate (ABA) displays renal FXR agonistic activity *in vitro* and FXR-dependent gene modulation *in vivo*. Treatment with ABA effectively diminishes renal inflammation, reduces apoptosis, and alleviates OS, thereby protecting mouse kidneys from IRI.

#### 4.5.5 Wedelolactone

Experimental data have indicated that wedelolactone (WED) can inhibit the proliferation of renal mesangial cells and safeguard renal podocytes (Shen et al., 2017). In summary, WED protects against inflammation and OS damage induced by doxorubicin in MPC-5 cells. The results (Zhu et al., 2019) demonstrated that WED alleviates doxorubicin-induced inflammation and OS damage to podocytes via the IκK/IκB/NF-κB pathway.

### 4.6 Other compounds

#### 4.6.1 Cordyceps sinensis extract

Cordyceps sinensis (CS) has been shown to reduce renal vascular resistance and enhance nephrotoxicity-induced renal dysfunction through antioxidant, anti-apoptotic, and anti-autophagic mechanisms (Wu et al., 2011). A recent study (Deng et al., 2020) revealed that the extract of C. cicadae mycelium regulates inflammatory responses in Cis-induced AKI model by inhibiting renal pathological alterations, inflammatory cell infiltration, and the release of various proinflammatory cytokines. It suggests that the C. cicadae mycelium extract exhibits significant anti-inflammatory properties, which are mediated through the inhibition of the TLR4/NF-κB/MAPK and HO-1/Nrf2 signaling pathways. Furthermore, the alleviation of cisplatin-induced nephrotoxicity attributed to the C. cicadae mycelium extract can, in part, be ascribed to the regulation of autophagy, the inhibition of apoptosis, and the upregulation of OAT expressions in kidney tissues. Li et al. (2024) found that CS extract 2′-deoxyadenosine mitigated AKI by improving renal pathophysiological alterations and inhibiting the expression of perforin and IFN-γ released from NK cells through the STING/IRF3 signaling pathway, thereby reducing damage to RTECs.

#### 4.6.2 Polygonum cuspidatum Sieb. et Zucc. Extracts

Polydatin (PD), a key component found in P. cuspidatum, has been shown to mitigate inflammation and OS in rats with AKI models. Additionally, emodin (Emo), another constituent of P. cuspidatum, has demonstrated the capacity to ameliorate AKI *in vivo* (Wang Y. et al., 2022). The domain of pharmacological research, which employs bioinformatics and network analysis, is emerging as a significant area referred to as NP. Collectively, the findings of this investigation (Yang et al., 2024b) suggest that PCE, along with its principal active constituents (Emo and PD), may confer protection against S-AKI through the attenuation of OS, inflammatory processes, and pyroptosis.

#### 4.6.3 Leontopodium leontopodioides extracts

Leontopodium leontopodioides (Willd.) Beauv (LLB) is primarily utilized in the management of both acute and chronic nephritis, urinary tract infections, proteinuria, and hematuria. Recent investigations have elucidated that LLB possesses anti-inflammatory, analgesic, diuretic, nephroprotective, and antioxidant properties (Gao Y. et al., 2019). Reports have indicated that LLB effectively inhibited the secretion of proinflammatory cytokines IL-6 and TNF-α in LPS-induced AKI. Moreover, LLB was found to downregulate NF-κB, p-PI3K, and pAKT, while simultaneously upregulating IκB expression, thereby providing renal protection against mesangial proliferative glomerulonephritis (MsPGN) in rat models (Zhao et al., 2019). Bai et al. (2023) noted that LLB holds promise in treating AKI via modulation of the NF-κB/NLRP3 signaling pathway.

#### 4.6.4 Hederasaponin C

Han et al. (2023) demonstrate that Hederasaponin C (HSC) inhibits the activation of the NLRP3 inflammasome through its interaction with the TLR4-regulated NF-κB and PIP2 signaling pathways. This discovery reveals previously unrecognized anti-inflammatory mechanisms of HSC, suggesting that its therapeutic strategy involving TLR4 modulation for AKI diverges from conventional clinical approaches such as dialysis and antibiotic therapies.

#### 4.6.5 *Achyranthes aspera* extract

Lin’s research (Lin et al., 2024) indicates that the water-soluble extract of *Achyranthes aspera* mitigates nephrotoxicity induced by cisplatin. This positive outcome can be partially ascribed to the activity of one of its active constituents, 20-hydroxyecdysone, which regulates various molecular signaling networks. Specifically, it downregulates genes and pathways related to DNA damage, OS, inflammation, and PANoptosis, while concurrently upregulating genes and signaling pathways associated with cell survival, including autophagy and mitophagy.

#### 4.6.6 Hazel leaf polyphenol extract

Sun et al.’s study (Sun et al., 2024) illustrated that cisplatin-induced ferroptosis leads to damage of renal tubular cells and renal dysfunction, which is associated with increased phosphorylation of yes-associated protein. The extract of hazel leaf polyphenols effectively mitigates AKI by inhibiting OS, apoptosis, and ferroptosis within the kidney, achieved through suppressing the Hippo signaling pathway.

#### 4.6.7 *Agathis robusta* Bark Extract

Recent findings (Mohamed et al., 2022) employing phytochemical analyses, *in silico* network modeling, docking techniques, and subsequent *in vivo* preclinical validation indicate that *Agathis robusta* Bark Extract (ARBE) may demonstrate renal protection by mitigating inflammation and apoptosis. The underlying mechanism is potentially associated with the downregulation of HSP90 and P53. Further investigations into drug discovery, alongside preclinical and clinical studies focusing on key components of ARBE, are recommended due to their predicted interactions with multiple targets, particularly the central hubs.

#### 4.6.8 Aframomum melegueta seeds extract

A recent study (Abdou et al., 2021) has provided compelling evidence regarding the involvement of Nrf2/HO1 and AMPK/SIRT1 signaling pathways in diclofenac-induced AKI. Additionally, the nephroprotective effects of AMSE against diclofenac-induced AKI have been demonstrated. These protective actions are believed to be mediated through its antioxidant, anti-inflammatory, and anti-apoptotic properties. Specifically, this involves the activation of the Nrf2 and AMPK/SIRT1 pathways alongside inhibiting NF-κB and STAT3 signaling.

#### 4.6.9 Ethanol extract of Illicium henryi

Recent research (Islam et al., 2019) demonstrated that the EEIH provides a favorable pharmacological intervention for preventing LPS-induced AKI, exhibiting significant anti-inflammatory and antioxidant activities. Pretreatment with EEIH has been found to improve renal pathological alterations, inflammatory responses, and oxidative/nitrosative stress. The therapeutic effects of EEIH are thought to be achieved by downregulation of the TLR4 and NF-κB pathways and upregulation of Nrf2 expression.

#### 4.6.10 Ferulic acid

Ferulic acid (FA), a widely distributed phytochemical and phenolic derivative of cinnamic acid, is predominantly found in the cell wall components of Angelica sinensis. It has been observed that FA exhibits anti-inflammatory properties by reducing production of inflammatory cytokines and release of ROS and RNS through the suppression of iNOS and COX-2, mediated by the activation of the NF-κB pathway (Lampiasi and Montana, 2016). Furthermore, FA has been shown to mitigate oxidative damage associated with sepsis by enhancing antioxidant capacity and reducing DNA damage in animal models subjected to cecal ligation and puncture (Bacanlı et al., 2014). Additionally, research has demonstrated the renal protective effects of FA against nephrotoxicity in various animal models (Bami et al., 2017). FA is a potent anti-inflammatory in acute and chronic inflammatory conditions (Sadar et al., 2016). A recent study (Mir et al., 2018) illustrated FA is a promising pharmacological intervention for preventing LPS-induced renal damage with minimal toxic effects. The protective mechanism of FA is believed to involve the downregulation of OS and inflammatory responses through the upregulation of Nrf2/HO-1 proteins and the inhibition of NF-κB signaling pathways.

#### 4.6.11 Tribulus terrestris L extract

The extract of Tribulus terrestris enhances RBF during the reperfusion phase via its vasodilatory effects, restoring the glomerular filtration rate to baseline levels (Najafi et al., 2014). This effect results in a relative improvement in plasma parameters indicative of kidney function. Furthermore, by reducing cellular damage and OS, Tribulus terrestris has the potential to inhibit the onset and progression of cellular injuries.

## 5 Strategies for SMFH and TCM phytochemicals globalization

The multi-targets and multi-levels regulation effects of TCM, are significant for various acute and chronic diseases. TCM is promoting the transformation of contemporary medical model from treatments of disease to the preventive treatments, from adversarial medicine to collaborative medicine, and from local medicine to holistic medicine. Researches on TCM are increasingly emphasizing the concept of translational medicine, adoption and compliance with standards and norms, which would make the researches more in-depth, quantitative and systematic.

The interaction of each component of TCM formulas are quantitatively analyzed, observed and studied by using internationally recognized methods and indexes. Finally, the mechanisms of action are illustrated scientifically, and researches’ papers are published in high-quality academic journals. It is required researchers to firmly grasp the three fundamental systems of *Chinese medicine resources, C. medicine quality, and clinical efficacy* to consolidate the scientific basis for the development of TCM. Modern medicine emphasizes the concept, norms and statistical analysis of evidence-based medicine. TCM should continue to explore and improve the efficacy evaluation techniques and methods that reflect its own characteristics and patterns, and strengthen the real-world research methods.

Actionable strategies advancing research, fostering global standardization, promoting interdisciplinary collaboration, and implementing robust clinical trials could be adopted. Measures such as optimizing the overall layout of TCM standard system, strengthening the supply of TCM standards in key fields, promoting the interactive development of TCM standards and scientific and technological innovation, promoting the internationalization of TCM standards, deepening the reform and innovation of TCM standardization, and consolidating the foundation for the development of TCM standardization are taken to achieve the standardization and internationalization of TCM, so as to integrate with mainstream medicine.

As mentioned previously, there are several preclinical/clinical studies demonstrating the short-term benefits of these SMFH and TCM phytochemicals. Previous research has revealed celastrol may alleviate inflammation and preclinical studies have confirmed its anticancer effects (Pan et al., 2023). Gastrodin injection has demonstrated positive treatment effects on dizziness or vertigo in clinic (Lai et al., 2022). The chemical composition of *A. robusta* Bark Extract (ARBE), depicted the interrelationship of the bioactive ingredients of ARBE with the I/R-AKI related molecular targets, and validated a nephroprotective effect (Mohamed et al., 2022). Ambiguous mechanisms of medical action, lacking purification procedures, relevant defective clinical ethical approval standards and low general recognitions, etc., are the barriers to translating these findings into clinical practice.

SMFH and TCM phytochemicals are promising therapeutic drugs for treating AKI. Although some TCM bioactive components have come into preclinical trials, it is essential to initiate preclinical pharmacologic and toxicologic researches to evaluate their efficacy and safety. In addition, it is widely recognized that modern medicine could relieve symptoms quickly while SMFH and TCM phytochemicals function therapeutic effects comprehensively (Li H. D. et al., 2019). In this regard, the combination of SMFH and TCM phytochemicals and modern medicine might become treatment strategies for AKI by taking advantages of both and limiting side effects.

## 6 Conclusion and perspectives

AKI is a clinical emergency condition. Western medicine treatment of AKI is still mainly symptomatic treatment, and with a further understanding of modern Chinese medicine for AKI, SMFH and TCM phytochemicals treatment of AKI has also achieved a certain effect. SMFH and TCM phytochemicals has gained increasing attention lately due to its plant-based sources and minimal adverse reactions, thus offering a promising avenue for addressing AKI. Early detection of the inducement of AKI, improvement of homeostasis, and avoidance of nephrotoxic drugs are helpful to the recovery of renal function. However, despite the unanimous recognition of the necessity of early recovery of AKI, studies on the treatment of AKI by SMFH and phytochemicals are still insufficient and lack uniform and objective syndrome differentiation standards. The results of this review clearly demonstrate that SMFH and TCM phytochemicals might alleviate AKI via multifunctional signal pathways and targets.

This review systematically elaborated the inducement factors of AKI and the potential mechanisms of various SMFH and TCM phytochemicals on AKI, providing evidences for the early diagnosis and preventive treatments of AKI, and offering a favorable basis for future experimental and clinical researches.
